# Supplementary material for: Strong and ductile titanium–oxygen–iron alloys by additive manufacturing
Source: Nature. 2023 May 31;618(7963):63–8. doi: 10.1038/s41586-023-05952-6 (PMC10232360; doi:10.1038/s41586-023-05952-6)
Supplement: Supplementary file 1 — This file contains Supplementary Figs. 1–20, Supplementary Tables 1–8, Supplementary Notes 1–7 and Supplementary References. [file 41586_2023_5952_MOESM1_ESM.pdf]

nature portfolio

https://doi.org/10.1038/s41586-023-05952-6

Supplementary information

Strong and ductile titanium–oxygen–iron alloys by additive manufacturing

In the format provided by the authors and unedited

Supplementary Information for

Strong and ductile titanium-oxygen-iron alloys by additive manufacturing

Tingting Song, Zibin Chen, Xiangyuan Cui, Shenglu Lu, Hansheng Chen, Hao Wang, Tony Dong, Bailiang Qin, Kang Cheung Chan, Milan Brandt, Xiaozhou Liao, Simon Ringer, Ma Qian

Correspondence to: ma.qian@rmit.edu.au; simon.ringer@sydney.edu.au

This file includes:

Supplementary Figs. 1 to 20

Supplementary Tables 1 to 8

Supplementary Note 1 β-flecks in titanium alloys and avoidance of Fe-stabilised β-flecks by DED

Supplementary Note 2 Chemical homogeneity, microstructure uniformity, porosity and predictability of *Simufact Welding* (DED)

Supplementary Note 3 Advantages of AM in producing the designed Ti-O-Fe alloys

Supplementary Note 4 Oxygen and iron in DED-fabricated Ti-O-Fe and Ti-6Al-4V alloys

Supplementary Note 5 The β-phase fraction in Ti-O-Fe alloys

Supplementary Note 6 Potential applications and implications

Supplementary Note 7 Repeatability of tensile stress-strain curves

DED: Directed Energy Deposition, which in this supplement refers to laser metal powder deposition.

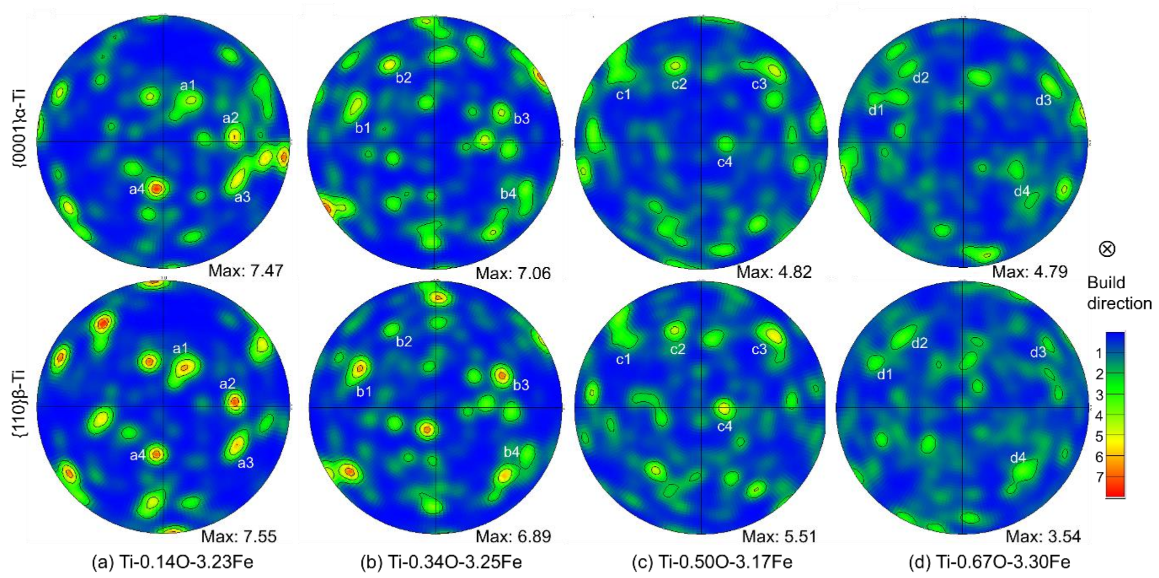

Supplementary Fig. 1 Normalised pole figures of α{0001} and β{110}, reconstructed from the EBSD IPF images in Fig. 1(d-g) of the article file (a total area of ~0.63 mm × 0.47 mm was analysed for each alloy). There exists an α{0001}//β{110} relationship for all alloys. The maximum value of the multiples of uniform distribution (MUD_{β}) for the prior-β grains ($\{110\}_{\beta}$) falls progressively from 7.55 to 3.54 with increasing oxygen concentration from 0.13% to 0.67%, indicative of a columnar-to-equiaxed transition (CET).

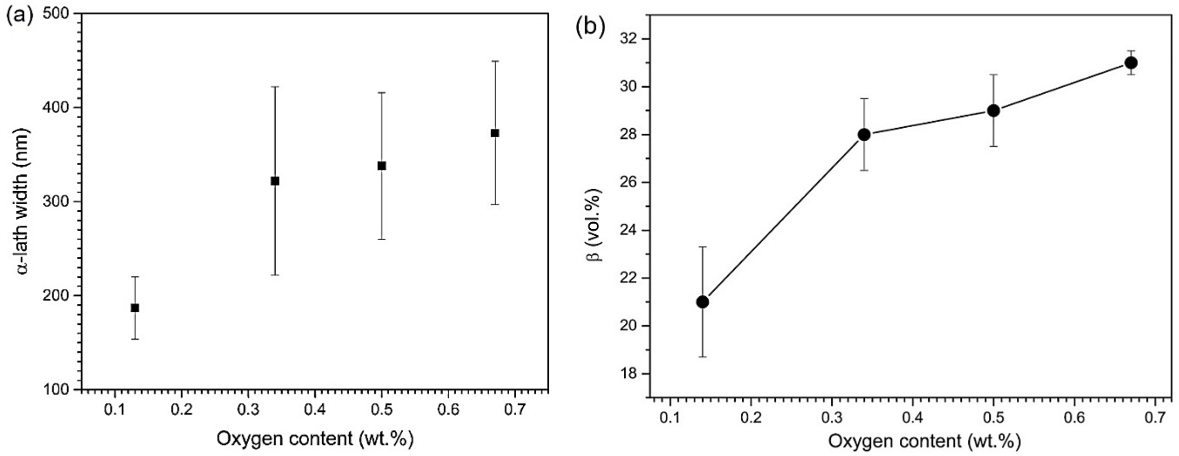

Supplementary Fig. 2 The influence of oxygen content on the microstructures of the Ti-O-Fe alloys printed in this work. (a) The mean α-lath width (300 measurements). (b) The mean β-phase fraction (measured from nine BSE images for each alloy).

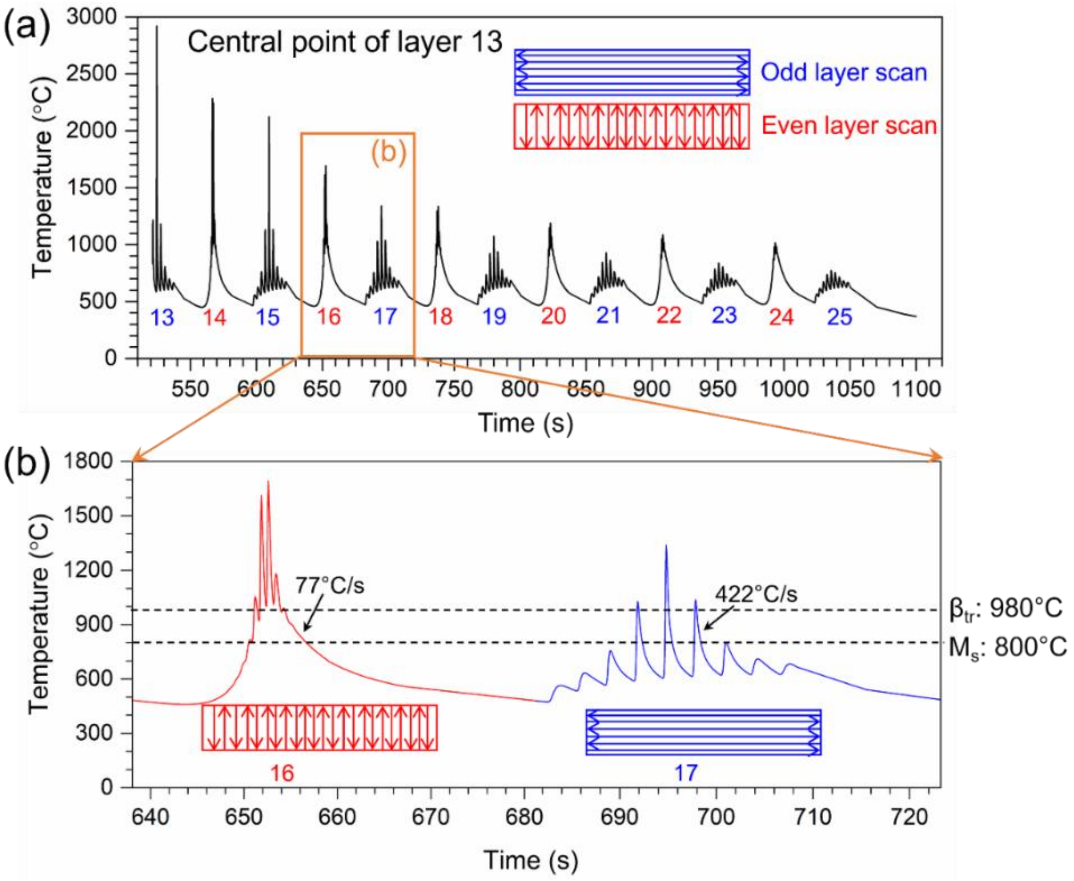

Supplementary Fig. 3 Bidirectional scan strategy for DED and the resulting thermal pulses (scan speed: 800 mm/min; layer interval: 15 s, see Extended Data Table 2 for other parameters). The sample has 25 layers. (a) Temperature evolution at the central point of layer 13 and the bidirectional scan strategy. The multiple peaks result from bidirectional scans. (b) A closer view of (a) from 630 to 735 s when layers 16 and 17 are deposited. β_{tr}: β transus; M_s: martensite formation temperature for Ti-6Al-4V. Each odd-number layer has nine scan paths. As a result, nine corresponding peaks or thermal pulses appear. Each even-number layer has 38 short scan paths. Since each short scan path is not thermal enough to result in a clear thermal pulse in previous layers, collectively, only a limited number of thermal pulses are observed from the deposition of layer 16.

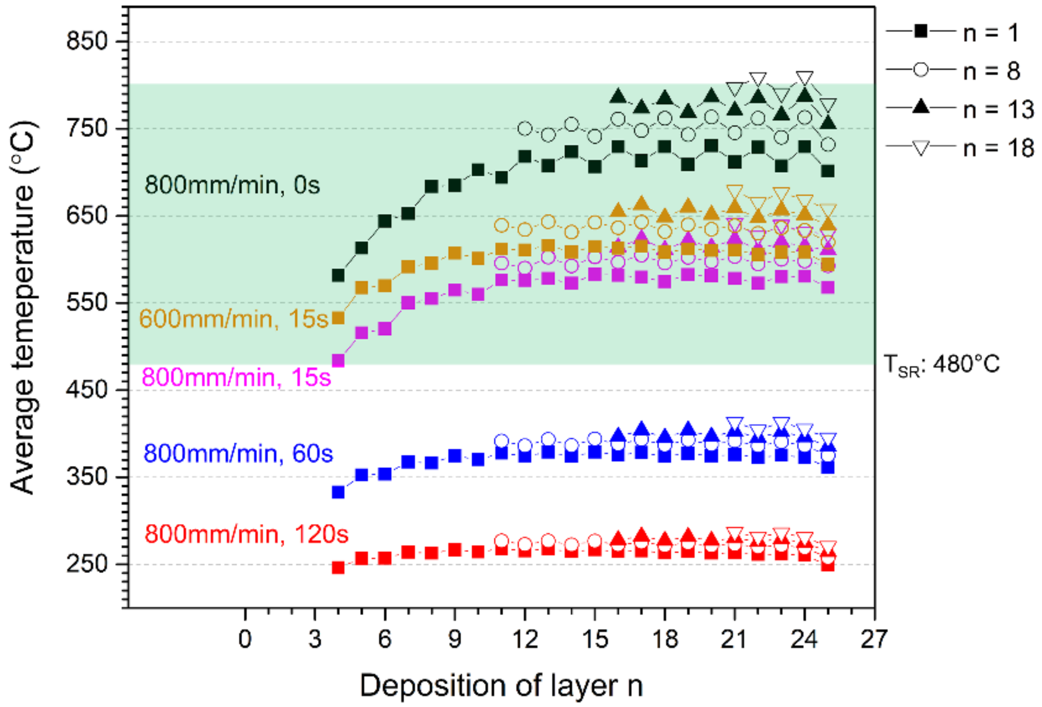

Supplementary Fig. 4 Evolution of the average temperature at the central points of layers 1, 8, 13 and 18 in the build during DED. All samples comprise 25 layers. Stabilisation occurs from the 8th or 9th layer of deposition. The stabilised temperature increases with reducing layer interval time or scan speed or with increasing build height.

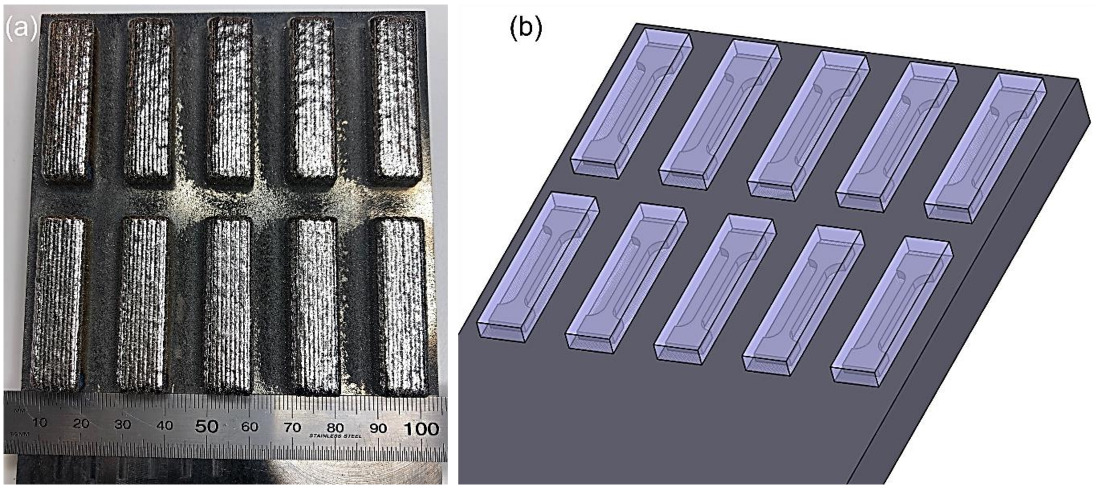

Supplementary Fig. 5 DED-printed coupons rectangular coupons (40 mm × 10 mm × 5 mm) at the layer thickness of 200 µm for tensile testing. (a) A photo of the printed coupons. (b) Schematic extraction of tensile specimens (12 mm × 3 mm × 2 mm as per Australian Standard AS 1391-2007) from the printed coupons. Each tensile specimen is extracted from the middle nine layers of the coupon. The temperature variation is within 30 °C across these nine layers by simulation. As a result, the microstructure is expected to be consistent. At least five coupons are printed for each condition.

Supplementary Table 1 Chemical compositions (wt.%) of important α–β Ti alloys developed to date[1,2].

(α-β)-Ti alloys	Al	Sn	Zr	Mo	V	Cr	Fe	Other
Ti-6-4	6				4			≤ 0.20O (Grade 5); ≤ 0.13O (Grade 23)
Ti-6-7	6							7Nb, ≤ 0.5Ta
IMI 550	6	2	4	0.5				
IMI 680	2.25	11	4	0.2				
ATI 425	4.5				3		1.8	0.3O
Ti-74	7			4				
Ti-6-6-2	6	2			6			0.7(Fe, Cu)
Ti-6-2-4-6	6	2	4	6				
IMI 551	4	4		4				0.5Si
Ti-6-2-2-2-2	6	2	2	2		2		0.25Si
Corona-5	4.5			5		1.5		
Ti-62S	6						2	0.1Si

Supplementary Table 2 The predicted β-transus temperature, α and β volume fractions, and O and Fe contents in the α and β phases in each Ti-xO-3Fe alloy and each Ti-6Al-4V-xO alloy at 650 °C using the CompuTherm (Pandat™) tool[3].

Composition (wt.%)	β transus, °C	α, vol.%	β, vol.%	O in α, wt.%	O in α, at.%	O in β, wt.%	O in β, at.%	Fe in α, wt.%	Fe in α, at.%	Fe in β, wt.%	Fe in β, at.%
Ti-0.14O-3Fe	918	77.98	22.01	0.180	0.537	8.0×10^{-4}	2.4×10^{-3}	0.32	0.27	12.30	10.73
Ti-0.34O-3Fe	975	78.74	21.26	0.434	1.287	1.5×10^{-3}	4.5×10^{-3}	0.23	0.19	12.97	11.33
Ti-0.50O-3Fe	1013	79.09	20.91	0.636	1.879	2.0×10^{-3}	6.1×10^{-3}	0.18	0.15	13.34	11.65
Ti-0.67O-3Fe	1058	79.87	20.13	0.845	2.485	2.2×10^{-3}	6.7×10^{-3}	0.14	0.11	13.95	12.20
Ti-6Al-4V-0.14O	997	80.04	13.85	0.174	0.518	4.2×10^{-3}	1.25×10^{-2}	-	-	-	-
Ti-6Al-4V-0.34O	1040	81.63	12.55	0.414	1.228	9.0×10^{-3}	2.69×10^{-2}				
Ti-6Al-4V-0.50O	1082	82.63	11.47	0.602	1.780	1.16×10^{-2}	3.47×10^{-2}				
Ti-6Al-4V-0.67O	1140	83.65	10.37	0.798	2.349	1.32×10^{-2}	3.95×10^{-2}				
Ti-6Al-4V (Grade 5, 0.2O)	1010	79.56	14.30	0.250	0.74	5.6×10^{-3}	1.67×10^{-2}	-	-	-	-
Ti-6Al-4V (ELI, 0.13O)	987	79.98	13.93	0.161	0.48	3.9×10^{-3}	1.16×10^{-2}	-	-	-	-

C, N and H are considered in all calculations at accepted limits (C: 0.08 wt.%, N: 0.05 wt.%, H: 0.012 wt.%).

Supplementary Table 3 DED conditions considered for establishing the processing window.

Average cooling rate when cooled from the molten state to 800 °C			
Manufacturing condition (scan speed – layer interval)	Dimensions of the 25-layer sample (length × width × thickness)	Sample position	Average Cooling rate
1200 mm/min – 15 s	40 mm × 10 mm × 5 mm	Centre of layer 1	3,868 °C/s
800 mm/min – 0 s	40 mm × 10 mm × 5 mm	Centre of layer 1	2,356 °C/s
		Centre of layer 13	818 °C/s
		Centre of layer 25	646 °C/s
800 mm/min – 15 s	40 mm × 10 mm × 5 mm	Centre of layer 1	2,432 °C/s
		Centre of layer 13	1,444 °C/s
		Centre of layer 25	1,404 °C/s
800 mm/min – 60 s	40 mm × 10 mm × 5 mm	Centre of layer 1	2,881 °C/s
		Centre of layer 13	2,224 °C/s
		Centre of layer 25	2,401 °C/s
800 mm/min – 120 s	40 mm × 10 mm × 5 mm	Centre of layer 1	3,131 °C/s
		Centre of layer 13	2,619 °C/s
		Centre of layer 25	2,739 °C/s
600 mm/min – 15 s	40 mm × 10 mm × 5 mm	Centre of layer 1	1,855 °C/s
		Centre of layer 13	1,016 °C/s
		Centre of layer 25	1,008 °C/s
400 mm/min – 15 s	40 mm × 10 mm × 5 mm	Centre of layer 1	1,116 °C/s
200 mm/min – 15 s	40 mm × 10 mm × 5 mm	Centre of layer 1	450 °C/s

Supplementary Table 4 Atom probe tomography (APT) results from the literature about the distribution of O and Fe in the α-phase (C_{α}) and β-phase (C_{β}) of Ti-6Al-4V manufactured by different methods ($C_{\alpha\text{-}O}$: O in α; $C_{\beta\text{-}O}$: O in β; $C_{\alpha\text{-}Fe}$: Fe in α; $C_{\beta\text{-}Fe}$: Fe in β).

**Manufacture method**	**$C_{\alpha\text{-}O}$** (at.%)	**$C_{\beta\text{-}O}$** (at.%)	**$C_{\beta\text{-}O}$/ $C_{\alpha\text{-}O}$**	**$C_{\alpha\text{-}Fe}$** (at.%)	**$C_{\beta\text{-}Fe}$** (at.%)	**$C_{\beta\text{-}Fe}$/ $C_{\alpha\text{-}Fe}$**	**Ref.**
Laser powder bed fusion (LPBF)	0.5	0.4	0.8	0.2	2.5	12.5	4
	0.9	0.3	0.33	0.2	3.3	16.5	
			~0.2			~70	5
			~0.4				
	0.5	0.09	0.18	0.05	2.8	56.0	6
Electron beam powder bed fusion (EB-PBF)	0.41	0.24	0.59	0.16	3.28	20.0	7
	0.78	0.23	0.29				8
	0.41	0.24	0.58	0.16	3.28	20.5	9
	0.42	0.20	0.48	0.16	3.34	20.9	
LPBF + heat treatment at 400 °C	0.4	0.1	0.25	0.1	5.0	50.0	4
LPBF + heat treatment at 530 °C	0.7	0.2	0.29	0.1	7.7	77.0	
Laser metal deposition	1.05	0.74	0.70				8
Thermo-mechanical reversals between 400-650 °C – 45 cycles	0.4	0.1	0.25	0.1	2.0	20.0	6
Thermo-mechanical reversals between 400-650 °C – 75 cycles	0.4	0.1	0.25	0.06	1.7	28.3	
Arc-melting + beta forging + alpha-beta forging + ageing	0.29 (wt.%)	0.11 (wt.%)	0.38	0.06 (wt.%)	3.91 (wt.%)	65.2	10
DED (Ti-6Al-4V-0.22O-0.20Fe, wt.%)	0.56	0.27	0.482	0.08	1.97	24.63	This study
	0.64	0.27	0.422	0.09	1.97	21.89	
DED (Ti-0.14O-3Fe, wt.%)	0.962	6.59×10^{-2}	0.068	0.0611	10.79	176.60	
DED (Ti-0.34O-3Fe, wt.%)	1.39	3.14×10^{-2}	0.023	0.0211	11.355	538.13	

Supplementary Note 1

β-flecks in titanium alloys and avoidance of Fe-stabilised β-flecks by DED

1.1 β-flecks in titanium alloys

Most information about the β-flecks in near-β Ti alloys is based on observations from billet or forged products. Assuming a uniform reduction during billet conversion, the width of β-flecks in commercial Ti alloy ingots is estimated to be 1-4.4 mm[11]. The length can be up to 10 times the width, i.e. up to a few centimetres. β-flecks form in the late-stage solidification when the solid fraction is greater than 0.8 or 0.9, calculated from the measured composition of the β-flecks using the Scheil model[12]. The distribution of the β-flecks is not uniform. Fig. 1 of Ref. [13] shows several cross-sectional views of the β-flecks (white patches) in a rolled and forged tensile sample of the Ti-10V-2Fe-3V alloy. The number density of the β-flecks (> 50 μm in size) is about 200/cm^2. Systematic experimental data revealed that these β-flecks reduce the tensile ductility of Ti-10V-2Fe-3V by more than 50% and the low cycle fatigue strength by more than 90% [13]. Large β-flecks are difficult to remove by annealing.

As already shown in Extended data Fig. 1, despite the high cooling rate, significant β-flecks (5.6%Fe) formed in the microstructure of the Ti-0.33O-3.11Fe alloy, fabricated by water-cooled copper mould casting (sample size: 120 mm × 12 mm × 5 mm, 32 grams, Methods). The number density of the β-flecks reached 400/cm^2. Together, this shows the high tendency for β-fleck formation in these Ti-O-Fe alloys through bulk solidification. Our calculations of the formation of the β-flecks (5.6%Fe), using the Clyne-Kurz model[14], by considering the back-diffusion of Fe and the actual cooling rate determined by the secondary dendrite arm spacing, predict their formation at the solid fraction of 0.85 for this particular alloy.

1.2 Avoidance of Fe-stabilised β-flecks in Ti-O-Fe alloys by DED

1.2.1 Cooling rate in Cu-mould casting and the formation of Fe-stabilised β-flecks

The formation of Fe-stabilised β-flecks arises from the excessive accumulation of Fe in the remaining liquid (often when the solid fraction $f_s \geq 0.8$ [12]) due to (i) the continuous rejection of the Fe atoms as solidification continues or f_s increases and (ii) the insufficient back-diffusion of Fe from the remaining liquid into the solid (i.e. the prior-β grains).

The solidification cooling rate $\dot{T}$ is an important and possibly decisive factor, as it may affect solute trapping depending on *the actual solid-liquid (S-L) interface velocity* V[15,16], where V is

related to the undercooling ΔT at the interface and a few other kinetic factors. Unfortunately, the relationship between ΔT and $\dot{T}$ has not been well established. In general, for small liquid volumes, it is convenient to use $\dot{T}$ because $\dot{T}$ can be measured or estimated directly. For example, for 75-1000 μm Cu-22Sn alloy droplets, the complete trapping of solute Sn occurred at $\dot{T} \geq 3.5\times10^4$ °C/s[17] (the decisive factor in this case). For the closely relevant Ti-1Al-8V-5Fe (Ti-185) alloy, it was found that plasma atomized spherical Ti-185 powders (50-150 μm) exhibited a uniform distribution of Fe (no β-flecks)[18], indicative of complete solute (Fe) trapping. The $\dot{T}$ of the 150 μm Ti-185 droplets is ~1.3×10^5 °C/s (estimated using Eqs. 3 and 4 of Ref.[19]). Again, the high $\dot{T}$ should be the decisive factor for the avoidance of β-flecks in this case because Ti-185 is prone to the formation of Fe-stabilised β-flecks in ingot metallurgy due to its 5%Fe.

Our Cu-mould cast Ti-0.35O-3Fe ingots were 5 mm thick. Experiments have shown that the $\dot{T}$ in the central region of a 5-mm diameter Cu-mould cast ingot is in the range of 10-80 °C/s (600-4800 °C/min) for both ferrous (Fe-25Ni) and non-ferrous (Al-33Cu) alloys[20,21], although the surface can reach 10^4 °C/s [20]. There were pores in the central region of our as-cast Ti-0.35O-3Fe alloy (Extended Data Fig. 1d). This provides a unique opportunity to study its $\dot{T}$. Supplementary Fig. 6 shows the dendritic prior-β grains observed in a pore cavity on the tensile fracture surface of this alloy. The secondary dendrite arm spacing (λ_2) is measured to be 13.83 ± 0.81 μm (excluding tertiary dendrites).

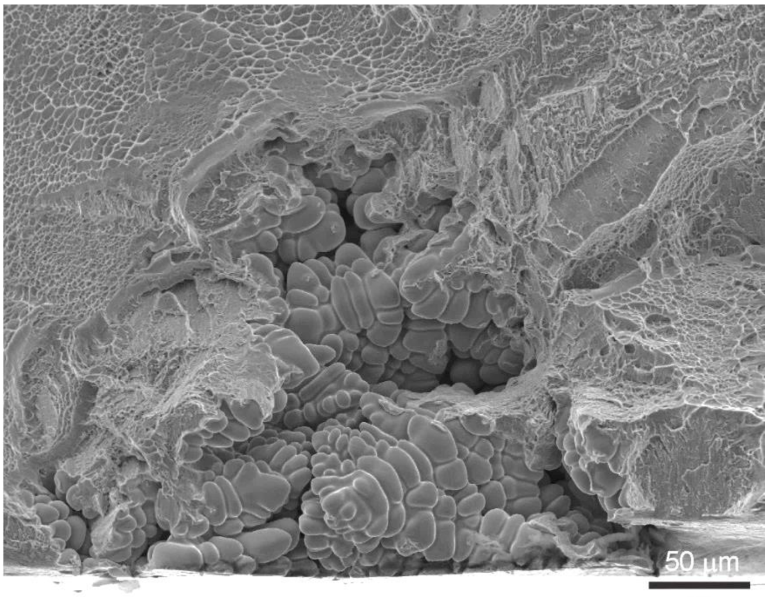

Supplementary Fig. 6 Dendritic prior-β grains in a pore cavity of the Cu-mould cast Ti-0.35O-3Fe alloy sample. The image is taken from a tensile fracture surface of the alloy.

An experimental relationship between the secondary dendrite arm spacing λ_2 and the solidification cooling rate $\dot{T}$ has been established for Ti-6Al-4V at cooling rates below 2430 °C/s[22]

$$\lambda_2 = 108.92\dot{T}^{-0.46} \quad (1)$$

As an approximate estimate, Eq. (1) predicts $\dot{T}$ = 89 °C/s for our Cu-mould cast 5-mm thick Ti-0.35O-3Fe ingot, close to the upper limit (80 °C/s) in the central region of the 5-mm thick Cu-mould cast ingot for both Fe-25Ni and Al-33Cu[20,21].

It is generally accepted that "For cooling rates up to 10^3 K/s, local equilibrium with compositional partitioning between the liquid and solid phases at the solidification interface is maintained. The interface undercooling is small. However, when the cooling rate increases above 10^3 K/s, nonequilibrium solidification occurs"[23], namely solute trapping occurs above 10^3 °C/s. Note that the Scheil equation or CALPHAD-numerical-Scheil is only applicable when the assumption of local equilibrium is valid. Therefore, at the cooling rate of $\dot{T}$ = 89 °C/s, no solute trapping is expected. Consequently, a significant accumulation of Fe is expected to occur in the remaining liquid. **This is the key reason** for the formation of the Fe-stabilised β-flecks in our Cu-mould cast Ti-0.35O-3Fe ingots.

It should be emphasised that the solidification of an ingot always starts from the mould walls and proceeds towards the centre. This sequential solidification process leads to continuous accumulation of Fe in the remaining liquid, conducive to the formation of β-flecks. This is *another* important contributing factor (melt volume is therefore important, which in turn affects the $\dot{T}$). Conversely, in the DED process, this cumulative effect is much weaker due to the small melt pool (1.08 mm^3), which corresponds to a much faster $\dot{T}$ (> ~2×10^3 °C/s, see below).

1.2.2 Approximate minimum $\dot{T}$ to help avoid β-flecks in the DED process used in this study

Before studying our Ti-O-Fe alloys, we first investigated the β-fleck issue in three binary Ti-Fe alloys: Ti-3Fe, Ti-5Fe and Ti-7Fe (all containing 0.13-0.14 O after DED). Supplementary Table 5 lists the predicted cooling rates in Ti-3Fe rectangular coupons built on a 10 mm-thick Ti-6Al-4V plate under different DED conditions. Each sample has 25 layers. Due to the lack of similar data for Ti-Fe alloys, we used the temperature-dependent thermophysical data of Ti-6Al-4V. Room temperature thermophysical property data cannot be used because these properties vary considerably between T_{room} and $T_{liquidus}$ (e.g. 4-6 times)[24].

As expected, $\dot{\boldsymbol{T}}$ decreases with increasing laser energy density but is influenced by the layer interval time. In each 25-layer build, the centres of the layers 18, 20 and 22 undergo slower cooling rates, with the slowest $\dot{T}$ being predicted to be **1994 °C/s**.

To evaluate the influence of the cooling rate on the formation of Fe-stabilised β-flecks, we printed rectangular coupons (40 × 10 × 5 mm^3; thickness: 5 mm) of Ti-3Fe, Ti-5Fe and Ti-7Fe alloys. Each composition was printed with three sets of DED conditions, listed in Supplementary Table 6. Each printed sample was examined layer by layer. No β-flecks were observed in any of these alloy samples.

Supplementary Table 5 Predicted solidification cooling rates at the centres of different layers in 25-layer builds (40 × 10 × 5 mm^3) when cooled from the third (last) remelting state to 1500 °C (solute enrichment lowers the $T_{solidus}$ of the Ti-3Fe alloy in the final liquid to ~1500 °C).

DED condition (laser power –scan speed – layer interval)	Laser energy density (J/mm^2)	Cooling rate at the centre of layer **n** (°C/s) – each sample has 25 layers					
		1	13	18	20	22	25
500 W–1200 mm/min–15 s	16.7	7054	6961	3251	3236	3527	12712
500 W–800 mm/min–60 s	25	7068	6507	3089	3074	3315	12551
500 W–800 mm/min–120 s	25	7211	6890	3223	3278	3478	13291
500 W–800 mm/min–0 s	25	6306	5311	2509	2492	2396	9981
500 W–800 mm/min–15 s	25	6151	5914	2796	2784	2729	11033
500 W–600 mm/min–15 s	33.3	5881	4968	2391	2462	2339	9695
500 W–400 mm/min–15 s	50	4532	4069	2080	2018	1994	6936

Supplementary Table 6 DED schedules for Ti-3Fe, Ti-5Fe and Ti-7Fe alloys.

Laser power (W)	Laser spot size (mm)	Traverse speed (mm/min)	Layer interval time (s)	Energy density E_d (J/mm^2)	Powder flow rate (g/min)	Step over (mm)	Overlap (%)
500	1.5	1200	15	16.7	1.7	1.05	70
500	1.5	800	15	25	1.7	1.05	70
500	1.5	400	15	50	1.7	1.05	70

*Carrier gas (He) flow: 10 L/min. Shielding gas (Ar) flow: 16 L/min.

We first discuss the Ti-7Fe alloy (Supplementary Fig. 7) and then briefly discuss the Ti-3Fe and Ti-5Fe alloys (Supplementary Fig. 8**)**. All micrographs were taken from around the 18th layer of each sample. The highest energy density (50 J/mm^2, v = 400 mm/min) produced the coarsest α-β

microstructure, coupled with acicular secondary α in the remaining Fe-containing β-phase (Supplementary Fig. 7**a-b**), due to the pronounced effect of the thermal cycles. The desired laser energy density (25 J/mm^2) resulted in ultrafine (~100 nm thick) α-β lamellae (Supplementary Fig. 7**c-d**). Unsurprisingly, the lowest energy density (16.7 J/mm^2, v = 1200 mm/min) entailed some defects (black dots in Supplementary Fig. 7**e-f**). Supplementary Fig. 8 shows a brief view of the microstructures of the Ti-3Fe and Ti-5Fe alloys printed under three DED conditions. At higher magnifications, they all consist of ultrafine (100-250 nm thick) α-β lamellae.

In summary, the DED conditions investigated in Supplementary Table 5 were all successful in avoiding the β-flecks, including in the Ti-7Fe alloy. All these DED conditions fall in the green zone of Fig. 1c. The **approximate slowest $\dot{T}$** identified by simulations for these alloy samples is **~2000 °C/s**.

Note that this approximate slowest solidification cooling rate (**~2000 °C/s)** and those predicted for the layers of 18, 20 and 22 in each sample (2000 – 3500 °C/s, Supplementary Table 5) are clearly slower than the cooling rates (10^4-10^5 °C/s) indicated earlier for complete solute trapping. Therefore, these cooling rates allow only partial solute (Fe) trapping to occur, reducing Fe accumulation in the remaining liquid, which helps avoid the formation of Fe-stabilised β-flecks.

As emphasized earlier, the small melt pool (1.08 mm^3) in the DED process limits Fe accumulation in the remaining liquid (f_s > 0.8) compared to ingot solidification. The actual accumulation of Fe is much weaker. This is another important factor to mitigate the formation of Fe-stabilised β-flecks.

Finally, the multiple thermal pulses and significant cyclic heating effects of the DED process, unlike conventional annealing, may decompose some of the Fe-rich β-phases. According to our literature review, this effect has not been investigated and in our opinion should not be neglected.

Therefore, we propose that the complete avoidance of Fe-stabilised β-flecks in these Ti-(3-7)Fe alloys may be the combined effect of the three factors mentioned above. *However, the prerequisite is the small melt pool, which determines the fast cooling rates (≥ 2000 ℃/s) and limited Fe accumulation in the remaining liquid (f_s > 0.8).*

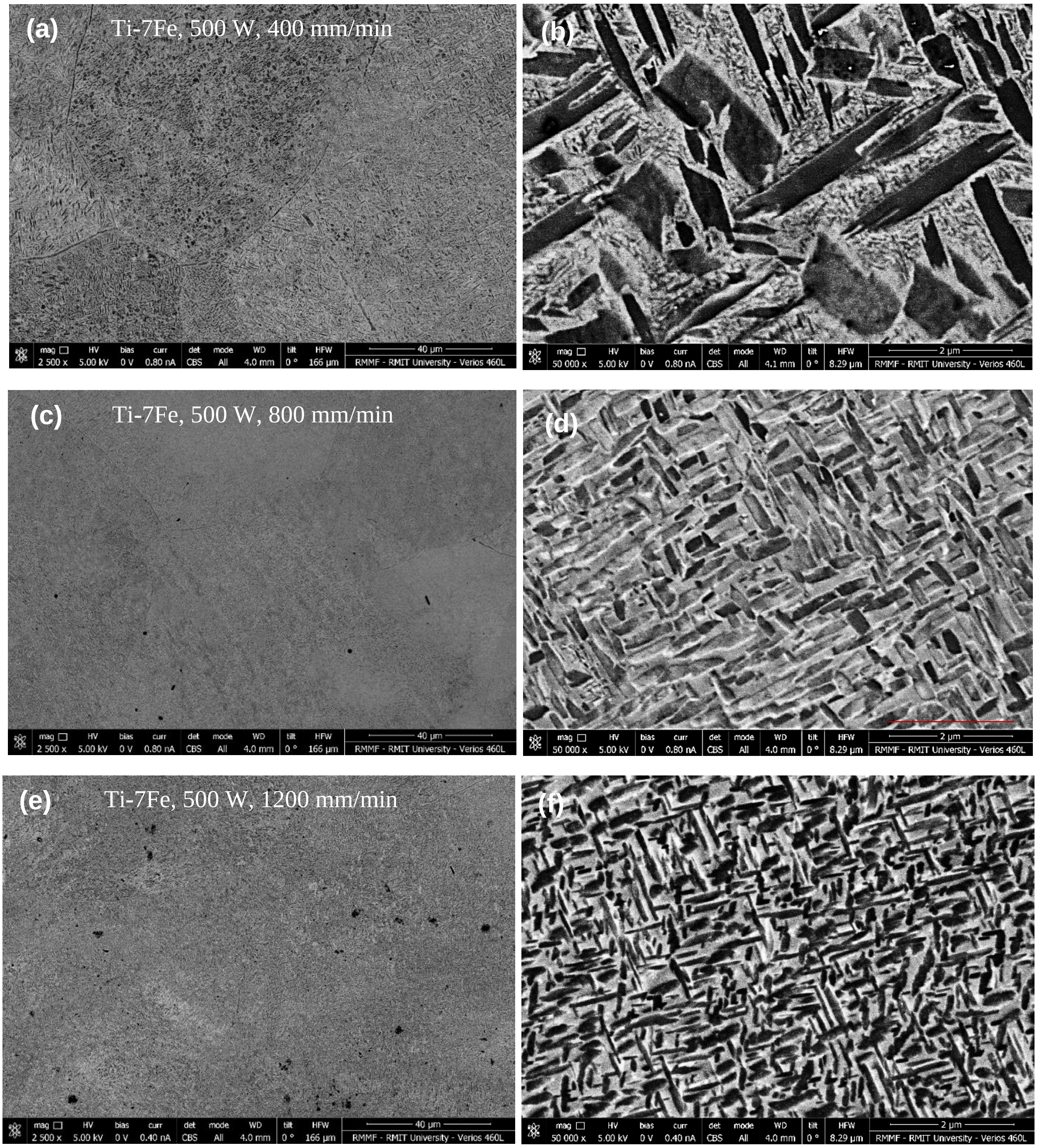

Supplementary Fig. 7 Backscattered electron images of the microstructures of the Ti-7Fe alloy printed under three sets of DED conditions. (**a, b**) 500 W, 400 mm/min, 50 J/mm^2. (**c, d**) 500 W, 800 mm/min, 25 J/mm^2. (**e, f**) 500 W, 1200 mm/min, 16.7 J/mm^2. (**a, c, e**) Low magnification. (**b, d, f**) High magnification. No β-flecks were observed along the build height of each sample layer by layer. The microstructure shown was observed from the region around the layer 18 of each sample, which exhibits approximately the lowest solidification cooling rate in each sample. No β-flecks were observed in any of these alloy samples.

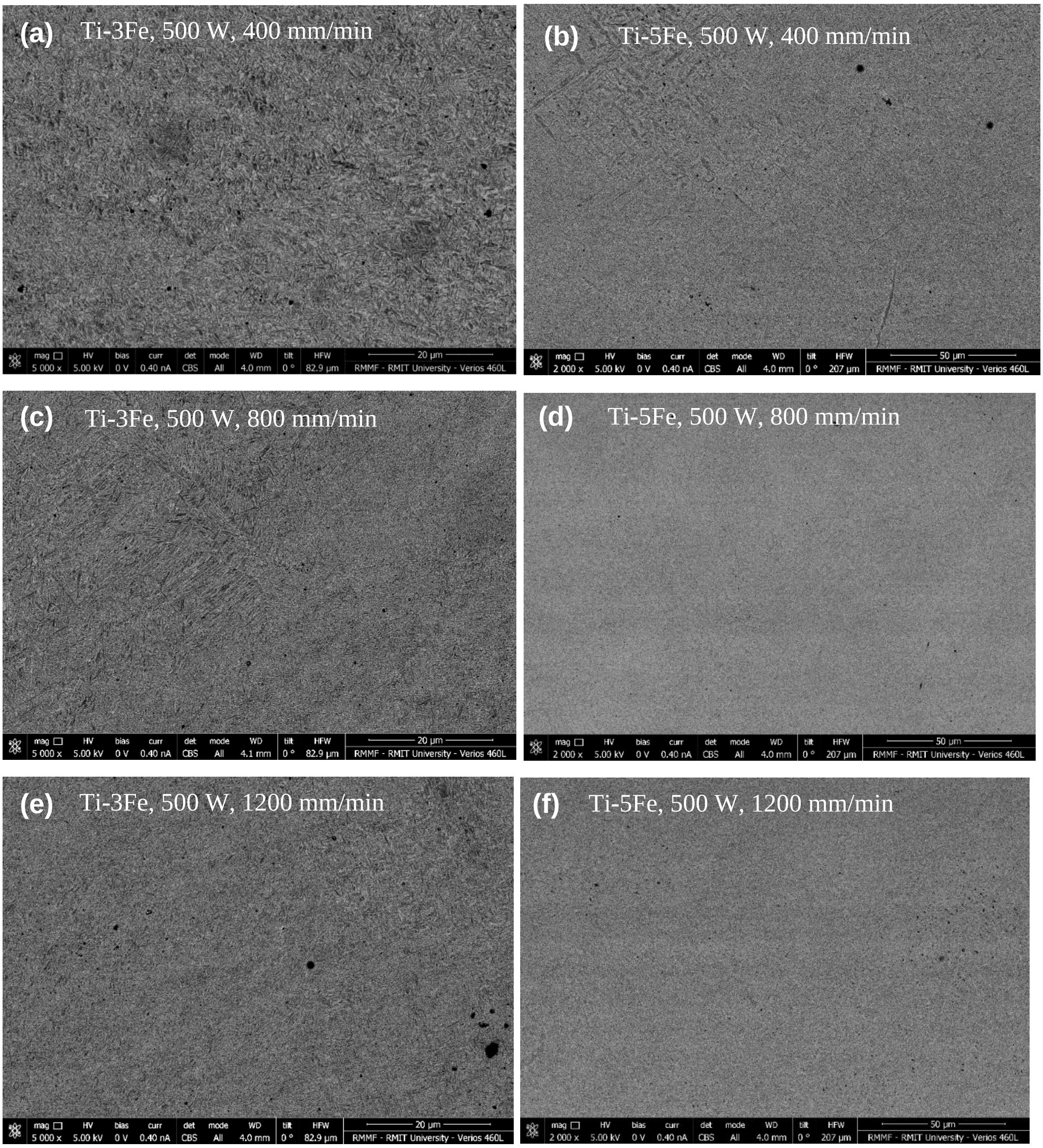

Supplementary Fig. 8 Backscattered electron images of the microstructures of the Ti-3Fe alloy (**a, c, e**) and Ti-5Fe alloy (**b, d, f**) printed under three sets of DED conditions. (**a, b**) 500 W, 400 mm/min, 50 J/mm^2. (**c, d**) 500 W, 800 mm/min, 25 J/mm^2. (**e, f**) 500 W, 1200 mm/min, 16.7 J/mm^2. The microstructure shown was observed from the region around the layer 18 of each sample, which exhibits approximately the lowest solidification cooling rate in each sample. No β-flecks were observed in any of these alloy samples. Most of the irregular black dots in (a) and (c) are not pores — they are coarser α-phase particles (see subsequent clarifications in Supplementary Fig. 14).

Supplementary Note 2

Chemical homogeneity, microstructure uniformity, porosity and predictability of Simufact Welding (DED)

2.1 Chemical homogeneity and microstructure uniformity

We used a laser spot size of 1.5 mm, which facilitates chemical homogeneity. Supplementary Fig. 9 shows half of a semi-ellipsoidal melt pool geometry resulting from the default DED conditions used in this work (laser power: 500 W; laser spot size: 1.5 mm; scan speed: 800 mm/min; layer interval time: 15 s). The melt pool volume at the centre of layer 13 is 1.08 mm^3 (major axis: 2.3 mm; minor axis: 1.5 mm; depth: 0.6 mm), where the Ti-0.35O-3.0Fe alloy has a liquidus temperature (T_L) of 1659 °C. This melt pool size is consistent with the literature data for similar DED conditions[25-27]. Each melt pool thus requires the melting of ~6,250 Ti powder particles in our study (D_v50: 69.09 μm).

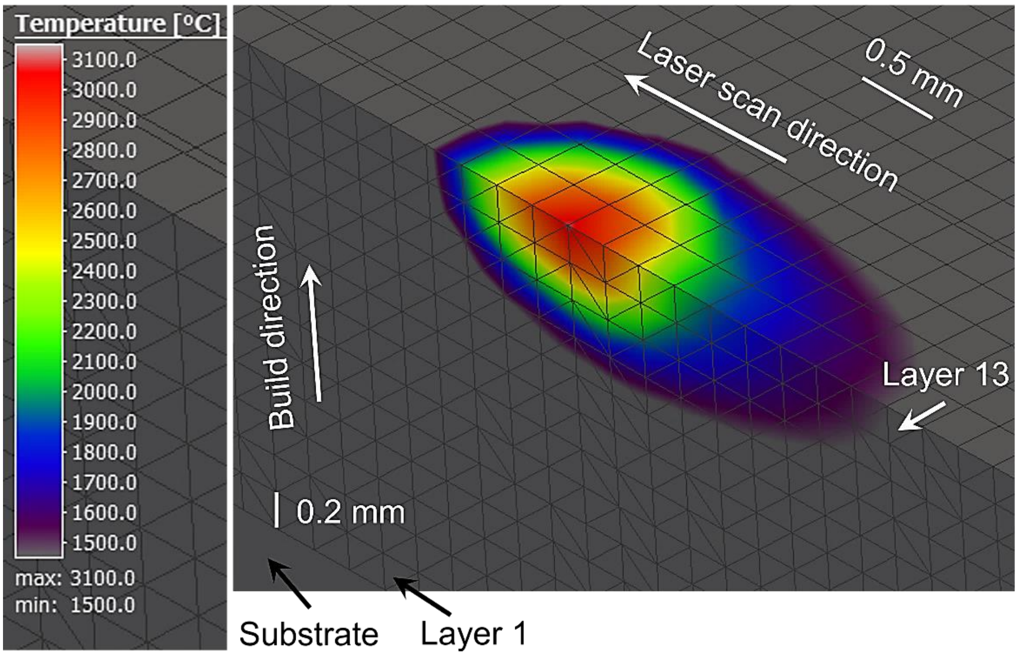

Supplementary Fig. 9 Melt pool geometry and volume at the centre of layer 13. Laser power: 500 W; laser spot size: 1.5 mm; scan speed: 800 mm/min, layer interval time: 15 s. The liquidus temperature (T_L) of Ti-6Al-4V is 1695 °C (T_L = 1658.5 °C for Ti-0.35O-3.0Fe). The resulting semi-ellipsoidal melt pool has a volume of 1.08 mm^3 (major axis: 2.3 mm; minor axis: 1.5 mm; depth: 0.6 mm). Each melt pool requires melting of ~**6,250** Ti powder particles with D_v50 = 69.09 μm.

Due to the high melt pool temperature (2000-3000 °C) and its dynamic nature, the composition within each small melt pool can quickly become homogeneous. For powder mixtures of only 2-3 substances or elements, chemical inhomogeneities are expected if the sample size is limited to a few hundred mixed powder particles. However, when the "sample" size exceeds 6000 mixed powder particles, it can be assumed that after a long mixing time, the average composition of each such "sample" (> 6000 powder particles) is consistent or varies within an acceptable narrow range. In addition, the melt pool depth is 0.6 mm (three layers). This means that each portion of the build will be remelted twice after the initial melting. This further helps to mitigate chemical inhomogeneity.

The above analyses were confirmed by our experimental measurements of the Fe content from different build heights shown in Supplementary Fig. 10. The measured compositions are consistent. Among all the samples we have fabricated, only a small batch-to-batch variation was observed in the Fe content from 3.12% to 3.36 wt.% (Extended Data Table 1). Commercial α-β Ti alloys normally allow for a much wider range of variations for each α or β stabilizer, e.g. Ti-6Al-4V (5.5-6.5%Al and 3.5-4.5%V) and ATI 425 (3.5-4.5%Al, 2.0-3.0%V, 1.2-1.8%Fe). The O content is even easier to control than the Fe content (Extended Data Table 1).

The chemical homogeneity is further supported by the very similar microstructures obtained from each batch of coupons fabricated under the same conditions. These observations are shown in Supplementary Figs. 11-12. Therefore, for simple low-alloy content (≤ 5wt.%) alloys, such as Ti-(0.35-0.50)O-3Fe, if the densities of the mixing powder particles are not substantially different, it is practical to fabricate these simple alloys from mixed powders using DED with a large laser spot size (1.5 mm). The resulting composition is consistent and within the expected specification requirements. Nonetheless, for critical components, the use of pre-alloyed powder may still be preferred, although a small batch-to-batch variation also occurs to pre-alloyed powder compositions.

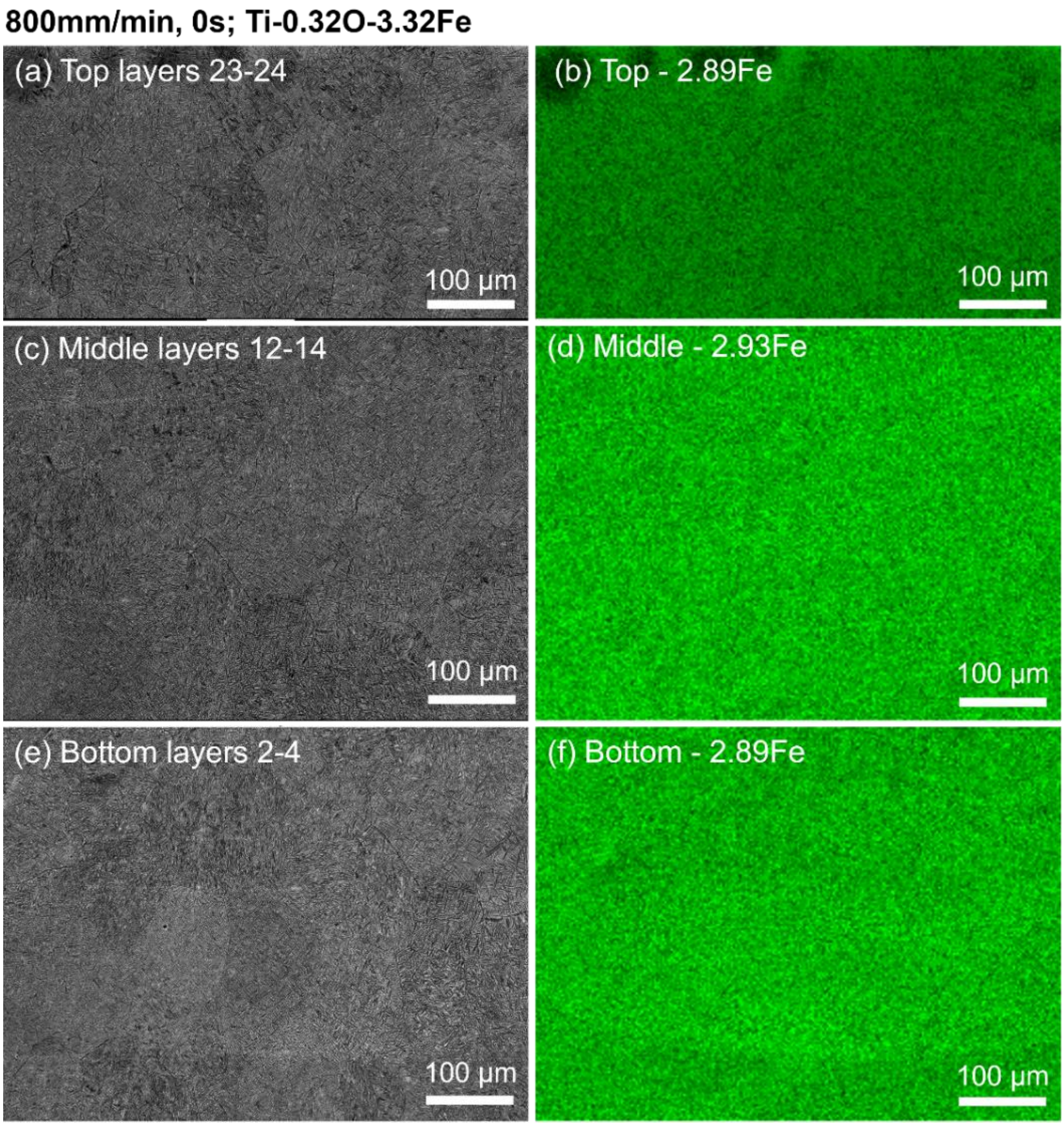

Supplementary Fig. 10 Microstructure (left column) and distribution of Fe (right column) at different build heights of the DED-fabricated Ti-0.32O-3.32Fe. The coupon comprises 25 layers. Scan speed: 800 mm/min; layer interval time: 0 s (see Extended Data Table 2 for other conditions). The distribution of Fe was obtained using energy dispersive spectroscopy (EDS). (a, b) Top layers 23-24. (c, d) Middle layers 12-14. (e, f): Bottom layers 2-4.

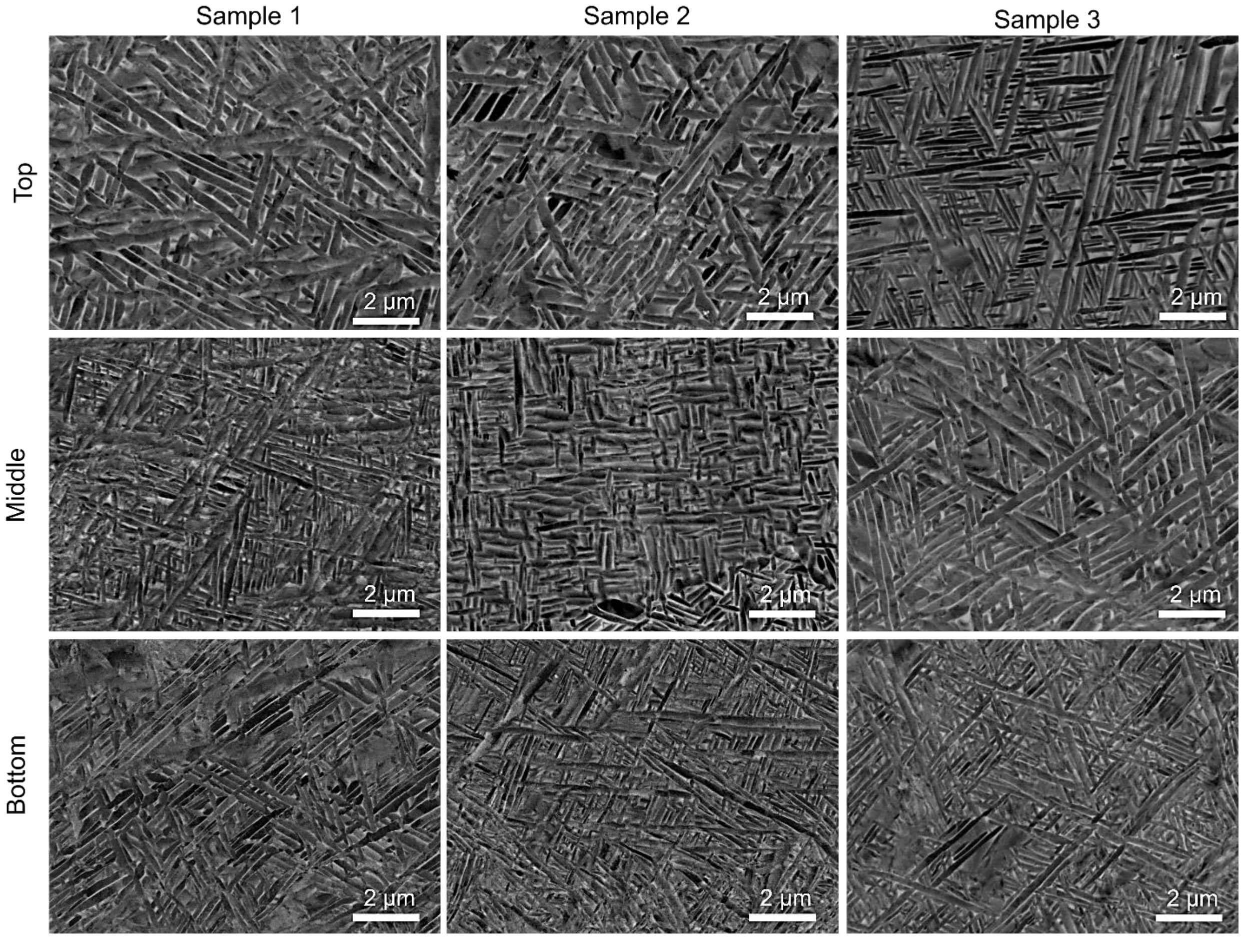

Supplementary Fig. 11 Comparison of the microstructures of three as-fabricated Ti-0.14O-3Fe tensile specimens along the build height (DED conditions: 800 mm/min; 15 s). The same batch of coupons have similar microstructures, consistent with the predictions based on simulation.

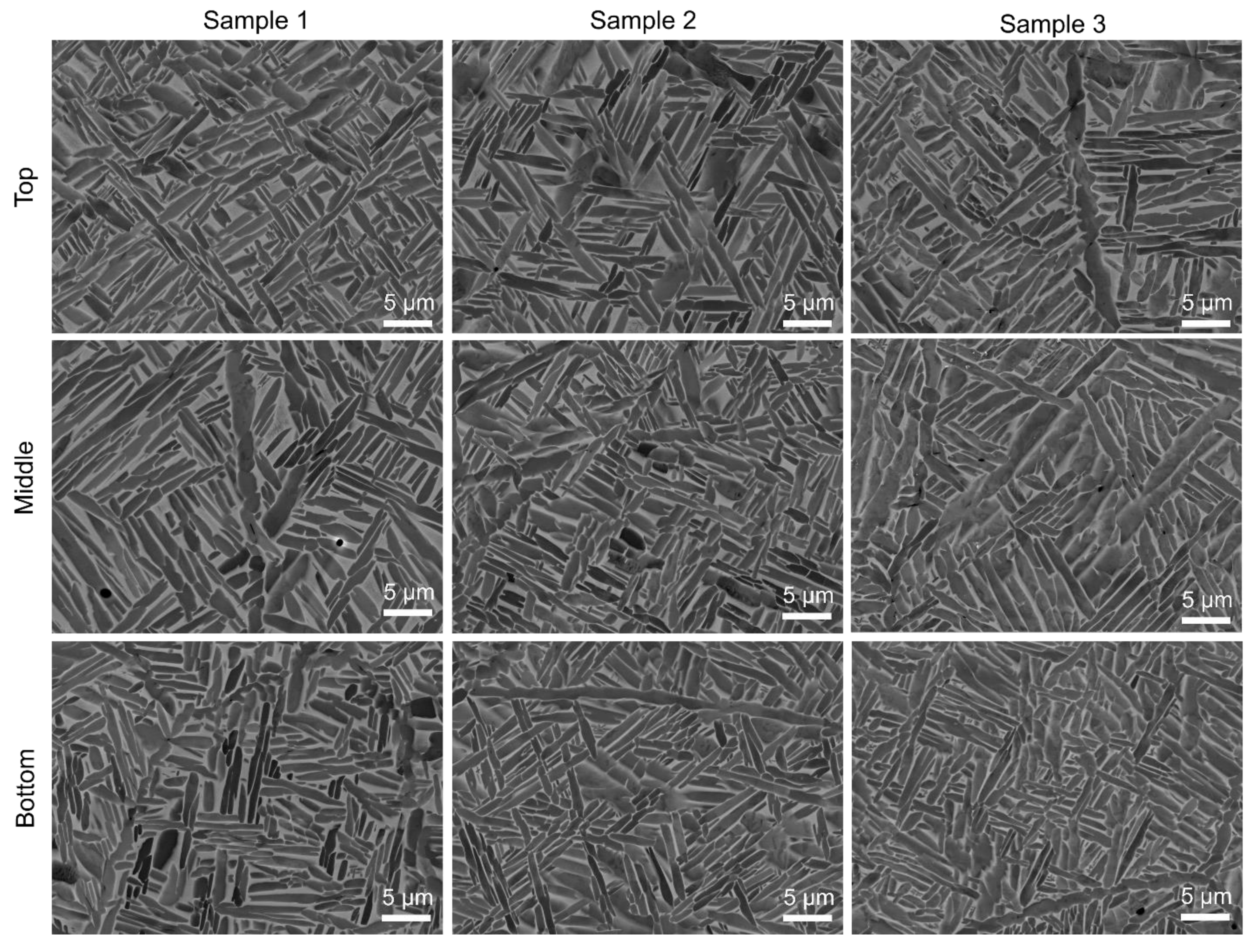

Supplementary Fig. 12 Comparison of the microstructures of three as-fabricated Ti-0.35O-3Fe tensile specimens along the build height (800 mm/min; 0 s). The same batch of coupons have similar microstructures, consistent with the predictions based on simulation.

2.2 Influence of porosity on tensile ductility

As previously indicated, our DED process (laser spot size: 1.5 mm; energy density: 25-35 J/mm^2; laser power: ≥ 500 W; overlap: 70%; powder feed: 1.7 g/min; layer thickness: 200 μm; layer interval: 0-15 s; scan strategy: bidirectional) consistently produces high-quality builds (Extended Data). Among all the printed Ti-0.35O-3Fe samples, the **lowest** tensile ductility (ε_f = 2.2 ± 0.6 %) was produced from 500 W – 800 mm/min – 120 s – 25 J/mm^2; the **highest** tensile ductility (ε_f = 21.9 ± 2.2 %) from 500 W – 800 mm/min – 0 s – 25 J/mm^2, and an **intermediate** tensile ductility (ε_f = 14.0 ± 0.7 %) from 500 W – 600 mm/min – 0 s – 33.3 J/mm^2. In terms of porosity, in fact, the Ti-0.35O-3Fe alloy samples with **the lowest porosity** and **smallest pores** exhibited the poorest ductility (ε_f = 2.2 ± 0.6 %), as demonstrated below.

Supplementary Fig. 13 shows a global view of the tensile fracture surfaces of these Ti-0.35O-3Fe samples with ε_f = 2.2 ± 0.6 % (**a**), ε_f = 14.0 ± 0.7 % (**b**) and ε_f = 21.9 ± 2.2 % (**c**). The number of discernible pores on each fracture surface is self-consistent and is typically ~5-6 only (red circles). This observation is similar for other samples reported in our Extended Data Fig. 2(a, b). Metallographically, pores were rarely observed in polished cross-sections, as exemplified in Supplementary Fig. 14 for the Ti-0.35O-3Fe alloy that exhibited the lowest ductility.

As Supplementary Fig. 13a confirms, five pores in the size range of 16-46 μm were randomly distributed on the entire tensile fracture surface of this lowest tensile ductility sample (ε_f = 2.2 ± 0.6 %). Compared to the same alloy samples in Supplementary Fig. 13 b-c, this sample has the least and smallest pores, but the lowest tensile ductility. Clearly, porosity is not the major factor controlling ductility here.

Compared with Supplementary Fig. 13a, seven larger pores in the size range of 45-72 μm were observed on the entire tensile fracture surface of the sample with ε_f = 14.0 ± 0.7 % (Supplementary Fig. 13b). However, the tensile ductility was six times higher (more pores with larger sizes). This reaffirms that porosity is not related to the substantially low ductility of the sample shown in Supplementary Fig. 13a.

The last sample (the same alloy) shown in Supplementary Fig. 13c exhibited the highest tensile ductility (ε_f = 21.9 ± 2.2 %). Six pores (red circles) in the size range of 20-65 μm were observed on the fracture surface. Similarly, there were more pores with larger sizes than those shown in Supplementary Fig. 13a. However, the tensile ductility of this sample was almost 10 times higher. Again, this indicates that porosity is not responsible for the substantially low ductility of the sample shown in Supplementary Fig. 13a. In fact, this high tensile ductility (21.9 ± 2.2 %) may suggest that spherical pores in the size range of 20-65 μm (small quantity) are not significantly detrimental to the tensile ductility of these titanium alloys at the strain rates tested here.

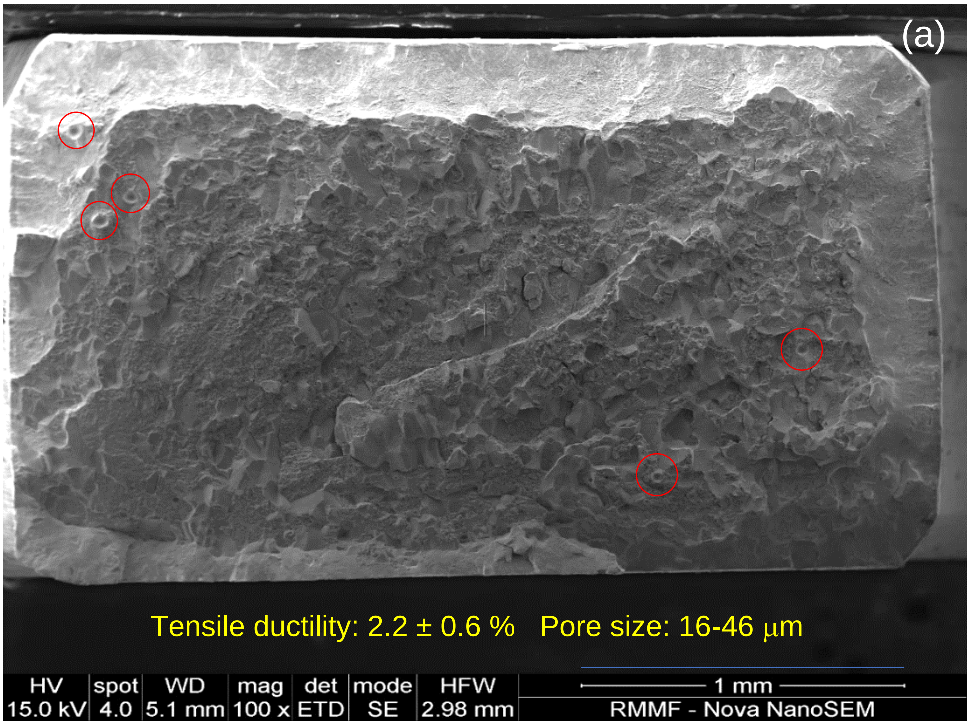
(a)
Tensile ductility: 2.2 ± 0.6 %   Pore size: 16-46 µm
HV 15.0 kV
spot 4.0
WD 5.1 mm
mag 100 x
det ETD
mode SE
HFW 2.98 mm
1 mm
RMMF - Nova NanoSEM

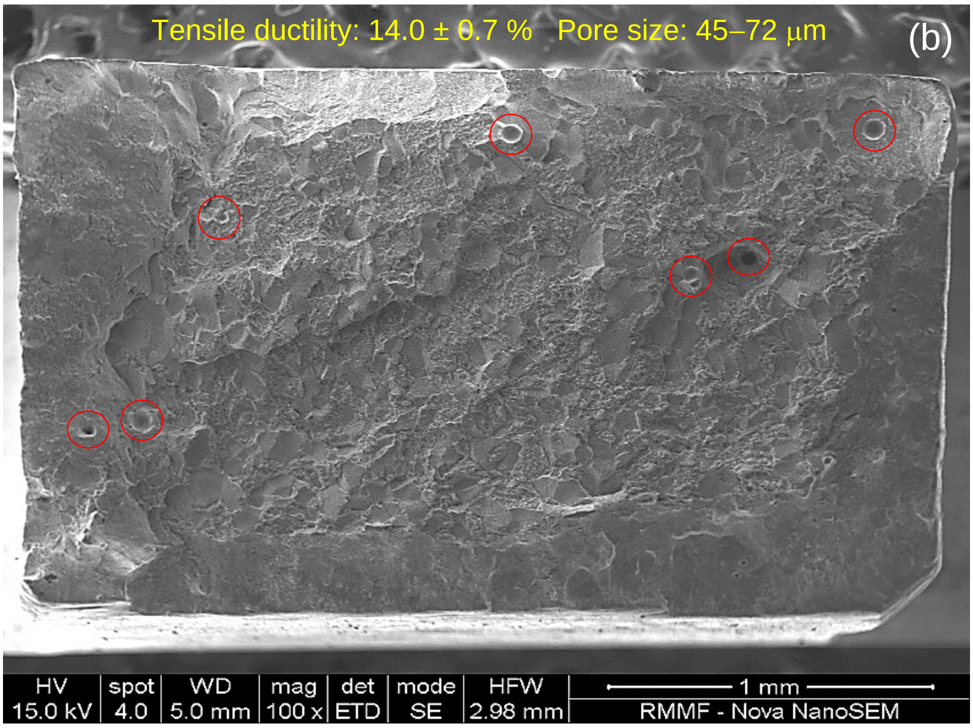
Tensile ductility: 14.0 ± 0.7 %   Pore size: 45–72 µm
(b)
HV 15.0 kV
spot 4.0
WD 5.0 mm
mag 100 x
det ETD
mode SE
HFW 2.98 mm
1 mm
RMMF - Nova NanoSEM

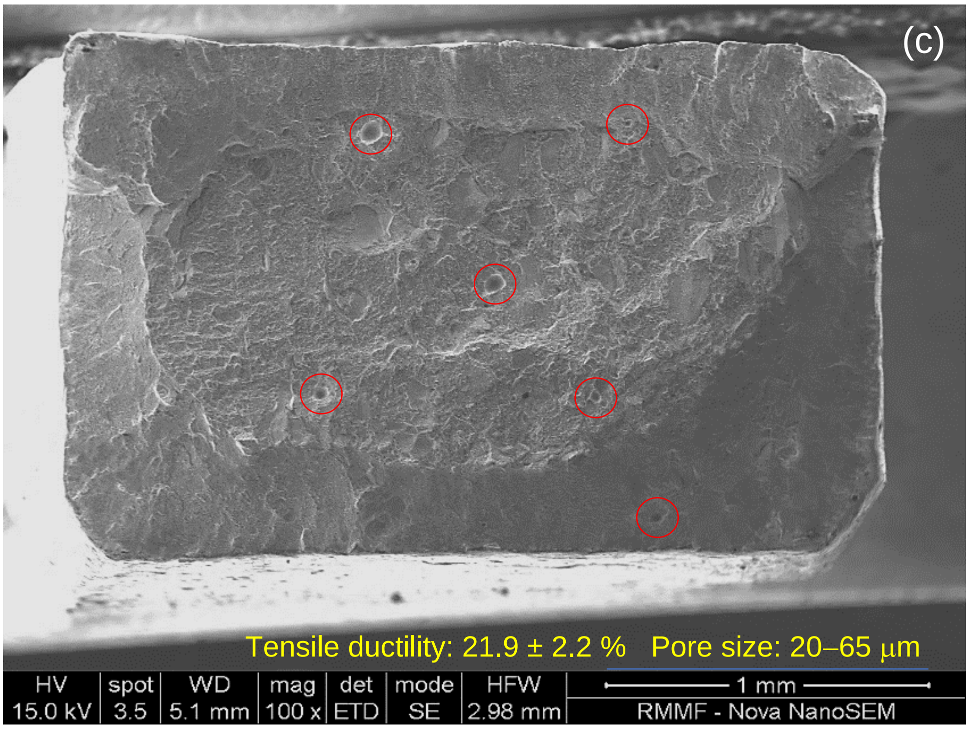

Supplementary Fig. 13 Influence of porosity on the tensile ductility of the Ti-0.35O-3Fe alloy printed under different DED conditions. (a) 500 W – 800 mm/min – 120 s – 25 J/mm^2; tensile ductility: 2.2 ± 0.6 %; pore size: 16-46 μm (five pores). (b) 500 W – 600 mm/min – 15 s – 33.3 J/mm^2; tensile ductility: 14.0 ± 0.7 %; pore size: 45-72 μm (seven pores). (c) 500 W – 800 mm/min – 0 s – 25 J/mm^2; tensile ductility (ε_f): 21.9 ± 2.2 %, pore size: 20-65 μm (six pores).

As mentioned earlier, our metallographic examinations rarely revealed any porosity in the microstructures of these Ti-O-Fe alloys deposited under the desired laser energy density conditions (25-35 J/mm^2). Supplementary Fig. 14a provides a low-magnification view of the as-manufactured microstructure of the Ti-0.35O-3Fe alloy, which exhibited the lowest tensile ductility (ε_f = 2.2 ± 0.6 %). No pores were observed. There were some irregular black dots, which are not pores but coarser α-phase particles, as shown in No pores were observed. There were some irregular black dots, which are not pores but coarser α-phase particles, as shown in Supplementary Fig. 14b (right edge).

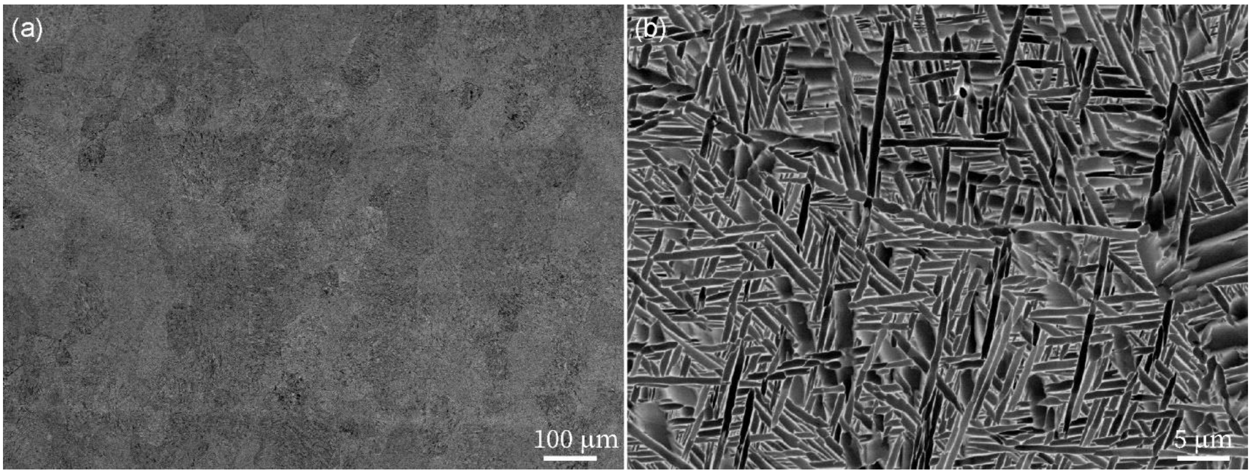

Supplementary Fig. 14 Metallographic examination rarely reveals any porosity in the Ti-0.35O-3Fe alloy printed under the desired DED conditions described in the work (E_d = 25-35 J/mm^2). Although this alloy exhibited the lowest tensile ductility (ε_f = 2.2 ± 0.6 %), it is not due to porosity. There are some irregular black dots in **(a)**, which are not pores, but coarser α-phase particles, as shown in **(b)** (right edge).

2.3 Predictability of the Simufact Welding (DED)

2.3.1 Melt pool shape and size

Before applying Simufact Welding (DED) to our Ti-O-Fe alloys, we systematically investigated the melt pool development by single-track DED experiments with Ti-6Al-4V under various conditions. Our Simufact Welding (DED) simulations are based on single-track DED experiments in order to properly define the heat source geometry (Extended Data Table 2) for our simulations. In other words, the basic input conditions for simulations are based on the single-track DED experiments obtained under various DED conditions.

As emphasized earlier, due to the lack of similar data for Ti-O-Fe alloys, we used the temperature-dependent thermophysical data of Ti-6Al-4V (the room-temperature data cannot be used because the thermophysical properties vary considerably between T_{room} and $T_{liquidus}$ (4-6 times)[22]. Therefore, to best assess the predictability of the Simufact Welding (DED) used for this study, we chose to compare the simulation results with the single-track DED experiments for Ti-6Al-4V.

As emphasized earlier, due to the lack of similar data for Ti-O-Fe alloys, the simulations used the temperature-dependent thermophysical data of Ti-6Al-4V (the room temperature data cannot be used because the thermophysical properties vary considerably between T_{room} and $T_{liquidus}$ (4-6

times)[22]. Therefore, to best assess the predictability of the Simufact Welding (DED) used for this study, we chose to compare the simulation results with the single-track DED experiments for Ti-6Al-4V.

Supplementary Fig. 15 compares the single-track DED Ti-6Al-4V melt pools (experimental) with the simulated melt pools for three DED conditions, 500 W – 400 mm/min (Supplementary Fig. 15a-b); 500 W – 800 mm/min (Supplementary Fig. 15c-d); and 500 W – 1200 mm/min (Supplementary Fig. 15e-f). The comparisons are quantified in Supplementary Table 7.

It is noteworthy that the density of Ti (ρ_{Ti}) increases profoundly when cooled from the melt pool temperature (up to 3100 °C by simulation for DED) to room temperature (RT, 4.51 g/cm^3). The authors of Ref. [28] reviewed the density measurements of molten Ti and experimentally determined the following relationship for the density of molten Ti up to 2127 °C (2400 K)

$$\rho_{Ti} = 4.14 - 2.15\times10^{-4}\,(T - T_m) - 3.71\times10^{-8}\,(T - T_m)^2 \qquad (2)$$

The latest study of the density of molten Ti (up to 1817 °C)[29] is consistent with Eq. (2).

Extrapolating Eq. (2) yields ρ_{Ti} = 3.756 g/cm^3 at 3100 °C (T_m = 1668 °C). This means a 20% increase in density when cooled to RT. Therefore, the simulated melt pool volume is expected to be at least 20% larger than that observed at RT. In other words, if the difference is within 20-30%, it should be considered highly consistent. Our Supplementary Fig. 15 and Table 7 confirm the high predictability (on the micron length scale) of the DED simulation module used in this work.

Supplementary Table 7 Experimental and simulated melt pools for Ti-6Al-4V ($T_{Solidus}$: 1604 °C) via a 40 mm long single track (the melt pool profile was taken from the middle of the track).

DED Condition	Melt pool (experimental)[a]			Melt pool (simulated)[b]		
	Width, µm	Height, µm	Penetration[c], µm	Width, µm	Height, µm	Penetration, µm
500 W 400 mm/min	1479	719	68	1640	803	74
500 W 800 mm/min	1202	469	108	1264	538	133
500 W 1200 mm/min	1073	351	125	1160	412	159

[a] Laser spot size: 1.5 mm; powder flow rate: 1.7 g/min; carrier gas (He) flow rate: 10 L/min; shielding gas (Ar) flow rate: 16 L/min.
[b] The heat source for simulation has a Gaussian parameter of 1 and an absorption efficiency of 35%.
[c] Penetration: the depth of the melt pool below the substrate surface
Substrate: a 200 mm × 100 mm × 12 mm Ti-6Al-4V plate for both experiments and simulations.

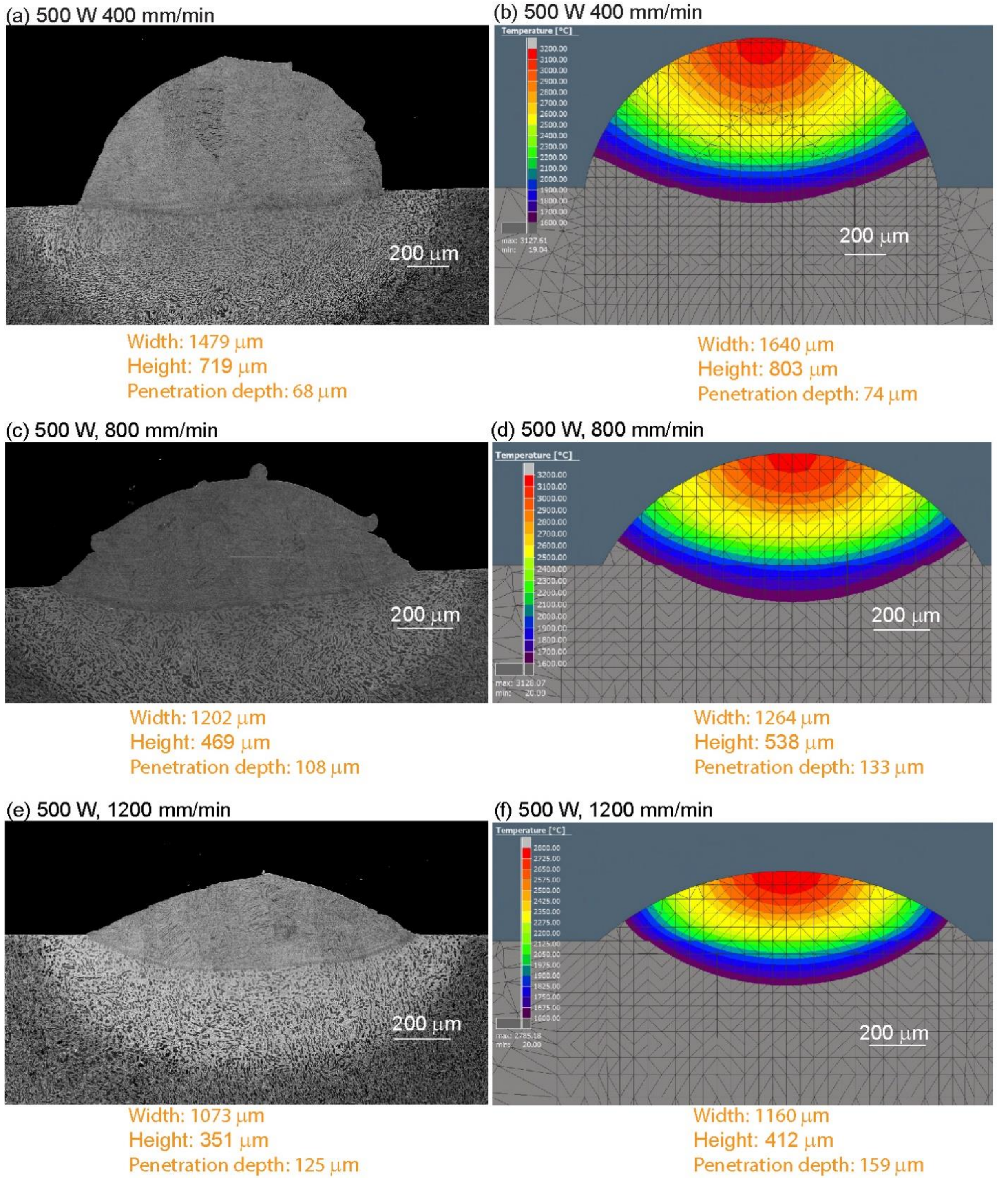

Supplementary Fig. 15 Experimental (single track DED) and simulated Ti-6Al-4V DED melt pools. (**a**) Experimental (laser power: 500 W, spot size: 1.5 mm, scan speed: 1200 mm/min). (**b**) Simulated (Simufact Welding DED). (**c**) Experimental (laser power: 500 W, spot size: 1.5 mm, scan speed: 800 mm/min). (**d**) Simulated (Simufact Welding DED). (**e**) Experimental (laser power: 500 W, spot size: 1.5 mm, scan speed: 400 mm/min). (**f**) Simulated (Simufact Welding DED).

2.3.2 THERMAL COOLING

The above assessment can be used as a valid evaluation of the predicted thermal cooling predictions. Direct and accurate measurements of the cooling rate (solidification or solid state) remain a challenge for a typical DED process due to the small melt pool and the layer additive manufacturing nature (**the location of interest does not pre-exist**).

An indirect assessment of the cooling rate for DED of Ti alloys is to use the relationship between the secondary dendrite arm spacing (λ_2) or the prior-β grain size (λ_1) and the solidification cooling rate $\dot{T}$. Unlike the Cu-mould cast ingots (Supplementary Fig. 6), no prior-β dendrites were observed in any DED-fabricated Ti-O-Fe alloy samples of this work (all being elongated or equiaxed prior-β grains). Therefore, we focus on the prior-β grain size λ_1.

Broderick et al.[19] have established that the prior-β grain size λ_1 (μm) of Ti-6Al-4V under rapid solidification can be described as a function of the solidification cooling rate $\dot{T}$ (K/s), i.e.

$$\lambda_1 = 3.1 \times 10^6 \, \dot{T}^{-0.93 \pm 0.12} \quad (3)$$

Eq. (3) was established for Ti-6Al-4V, which may need to be modified for our Ti-O-Fe alloys, because the observed prior-β grains are much finer than the prior-β grains of Ti-6Al-4V under the same DED conditions. This implies that the exponent of $\dot{T}$ in Eq. (3) should be greater than 1.05 (0.93 + 0.12). By applying Eq. (3) to the DED-fabricated binary Ti-3Fe alloy samples shown earlier (Supplementary Fig. 8) using the predictions of $\dot{T}$ in Supplementary Table 5, the exponent of $\dot{T}$ was found to be around 1.15. Therefore, we used the following Eq. (4) to further evaluate the solidification rate $\dot{T}$ of our Ti-O-Fe alloys studied in this work and then compare the results with Simufact Welding (DED) predictions:

$$\lambda_1 = 3.1 \times 10^6 \, \dot{T}^{-1.15} \quad (4)$$

We measured the prior-β grain size in the surface layer of the Ti-0.35O-3Fe alloy samples printed under four sets of DED conditions. Representative prior-β grain structures observed in the surface layer of these samples are shown in Supplementary Fig. 16 for each selected DED condition, while Supplementary Table 8 summarises the predictions and measurements.

The equiaxed prior-β grains are not uniform in the surface layer of each sample, Supplementary Fig. 16, featured by a large standard deviation. We therefore focused on the mean prior-β grain size. As shown in Supplementary Table 8, in each case, the prior-β grain size obtained from Eq.

(4) based on the predicted cooling rates matched well with the measured mean prior-β grain size on the micron length scale, reaffirming the good predictability of the Smiufact Welding (DED).

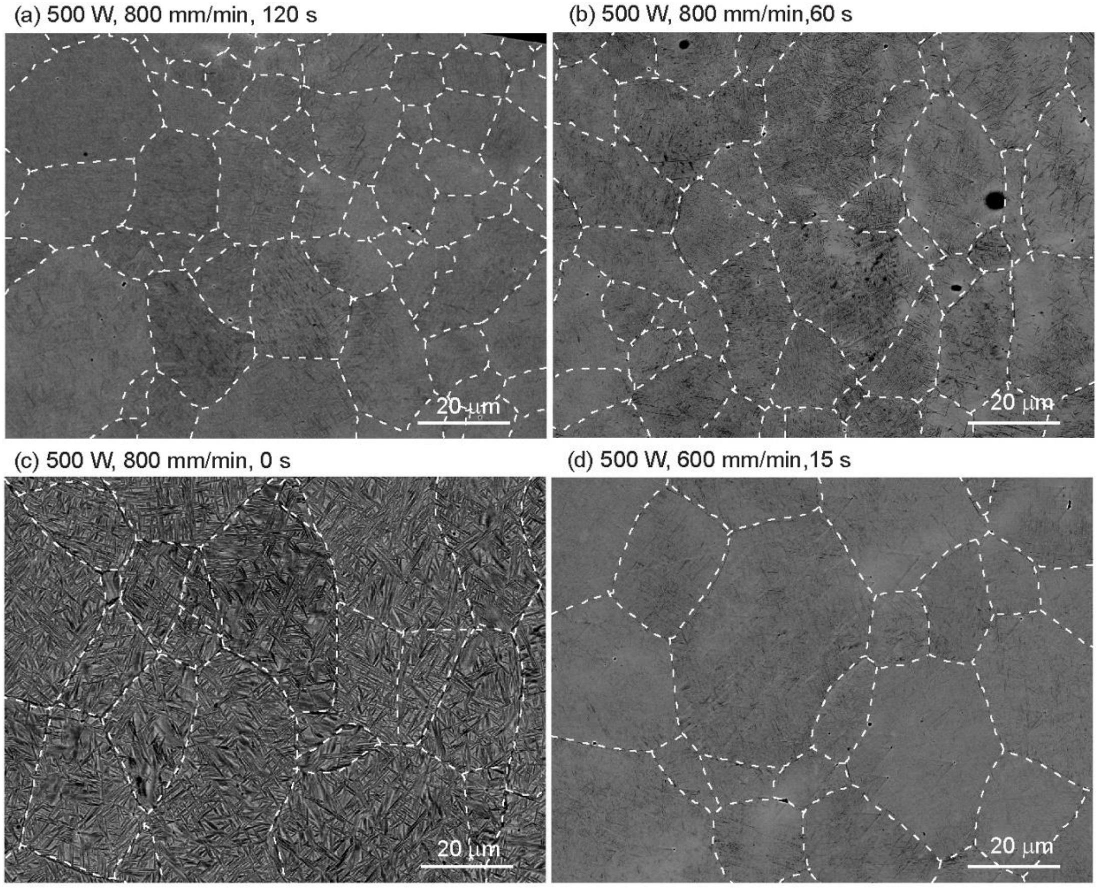

Supplementary Fig. 16 Prior-β grain structures observed in the surface layer of each sample of the Ti-0.35O-3Fe alloy printed under different DED conditions. (a) 500 W – 800 mm/min – 120 s. (b) 500 W – 800 mm/min – 60 s. (c) 500 W – 800 mm/min – 0 s. (d) 500 W – 800 mm/min – 15 s. Other DED parameters are listed in Extended Data Table 2.

Supplementary Table 8 Further assessment of the predictability of Simufact Welding DED — predicted prior-β grain size using Eq. (4) vs. measured mean prior-β grain size in the top surface layer of Ti-0.35-3Fe samples printed under four DED conditions.

DED condition	Predicted surface cooling rate $\dot{T}$ in °C/s	Predicted surface cooling rate $\dot{T}$ in K/s	Predicted prior-β grain size using Eq. (4) (μm)	Measured prior-β grain size (μm)
500 W-800 mm/min-120 s	13291	13564	55	41 ± 20
500 W-800 mm/min-60 s	12551	12824	59	64 ± 30
500 W-800 mm/min-0 s	9981	10254	76	75 ± 43
500 W-600 mm/min-15 s	9695	9968	78	76 ± 35

Supplementary Note 3

Advantages of AM in producing the designed Ti-O-Fe alloys

Compared with ingot metallurgy-based manufacturing techniques and shape casting, the DED (laser powder) process investigated offers the following advantages:

- Complete avoidance of the β-flecks in these Ti-O-Fe alloys, thereby enabling the use of Fe as the principal β-Ti stabiliser with desired microstructural homogeneity
- Advantage of free form fabrication (F^3) of large builds (near-net shapes)
- Advantage of in-situ formation of fine (350 nm thick) and short (8 μm long) α−β lamellar whiskers or structures, resulting in the strong but highly ductile α−β Ti-O-Fe alloys. For example, the DED-fabricated Ti-0.35O-3.0Fe achieved ε_f = 21.9 ± 2.2% and σ_{UTS} = 1034 ± 9 MPa, despite the high O content. This even meets the requirement for the tensile ductility of the mill-annealed commercially pure Ti (CP-Ti) Grade 2 ($\varepsilon_f \geq 20\%$, $\sigma_{UTS} \geq 345$ MPa), which is limited to O ≤ 0.25%[30].
- Capability and flexibility of tuning mechanical properties within a broad processing window or along the build height or across the wall thickness through adjusting the scan speed and/or layer-to-layer interval time.

Sintering-based net-shape or near net-shape powder metallurgy (PM) processes, including conventional powder metallurgy (C-PM), metal injection moulding (MIM) and hot isostatic pressing (HIP), can all avoid β-fleck formation by using either elemental powder blends (for C-PM) or pre-alloyed spherical powder (for MIM and HIP). However, the high temperature pressureless sintering process (1200-1300°C for 1-4 hours) for C-PM and MIM usually leads to high residual porosity (~2 vol.%), coarse β grains (due to long isothermal holding), and coarse α−β lamellae (due to the subsequent slow furnace cooling). Consequently, the resulting tensile mechanical properties are usually only comparable to their as-cast counterparts.

HIP is a possible option but not comparable to AM in terms of net shape formation. In addition, our HIP experience with Ti-6Al-4V indicates that even with a low HIP temperature (820 °C) and a high HIP pressure (200 MPa), HIP still yields very thick α-laths (~2 μm thick), resulting in low tensile strengths. Therefore, AM is the best net-shape fabrication method for these alloys which can also ensure excellent or outstanding tensile properties.

Supplementary Note 4

Oxygen and iron in DED-fabricated Ti-O-Fe and Ti-6Al-4V alloys

Supplementary Table 4 summarises the literature APT data on the partition of O and Fe in α and β phases in α–β Ti-6Al-4V manufactured by different processes, compared to the partition in two α–β Ti-O-Fe alloys printed in this work. Supplementary Fig. 17 plots their distributions. It is common for the β-phase in Ti-6Al-4V to contain about 40-80% of the O level in the α-phase, irrespective of the manufacturing method. This is fundamentally different from the virtually O-free β-phase detected in our printed alloys.

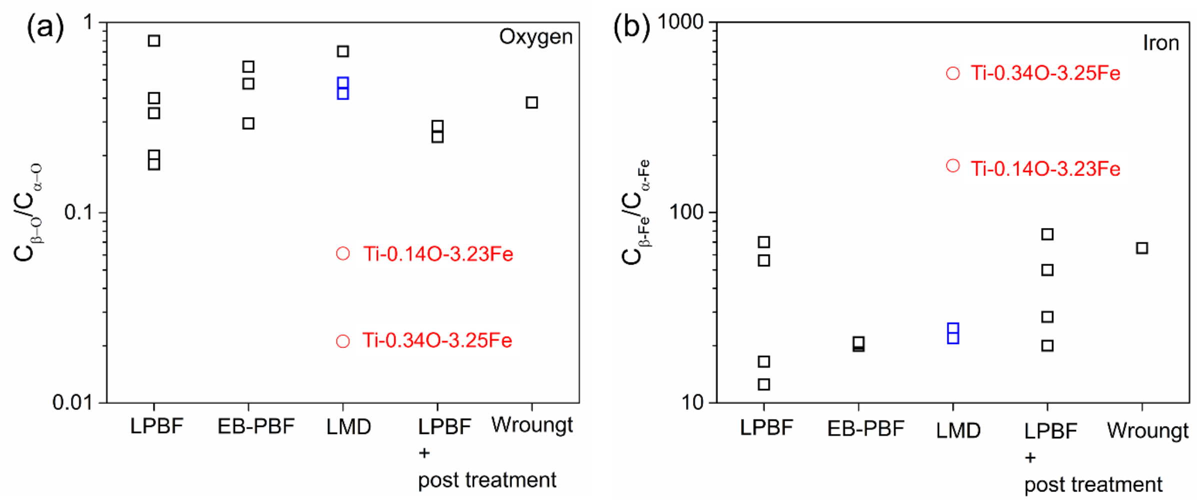

Supplementary Fig. 17 APT data on the partition of O and Fe in α and β phases in α–β Ti-6Al-4V manufactured by different processes, compared to the partition in the two α–β Ti-O-Fe alloys printed in this work. (a) O and (b) Fe. The two blue squares are for the reference Ti-6Al-4V printed in this work. Data source: Supplementary Table 4. $C_{α-O}$: O in α; $C_{β-O}$: O in β; $C_{α-Fe}$: Fe in α; $C_{β-Fe}$: Fe in β.

Supplementary Note 5

The β-phase fraction in Ti-O-Fe alloys

Increasing the O content resulted in an increase in the β-phase fraction in our Ti-O-Fe alloys (Supplementary Fig. 2). This is due to the partitioning effect of O and Fe between the α and β phases. For example, at 800 °C, CALPHAD indeed predicts that the Ti-0.14O-3Fe alloy contains more β-phase (59.8 vol.%β) than the Ti-0.67O-3Fe alloy (39.4 vol.%β), as shown in Supplementary Fig. 18. However, the lower the β-phase fraction, the higher the average Fe content in the β-phase and the more stable the β-phase will be. In this case, the average Fe content of the β-phase in the Ti-0.67O-3Fe alloy is 7.30%Fe vs. 4.85%Fe in the Ti-0.14O-3Fe alloy. This leads to a higher retained β phase fraction in the Ti-0.67O-3Fe alloy when cooled to room temperature.

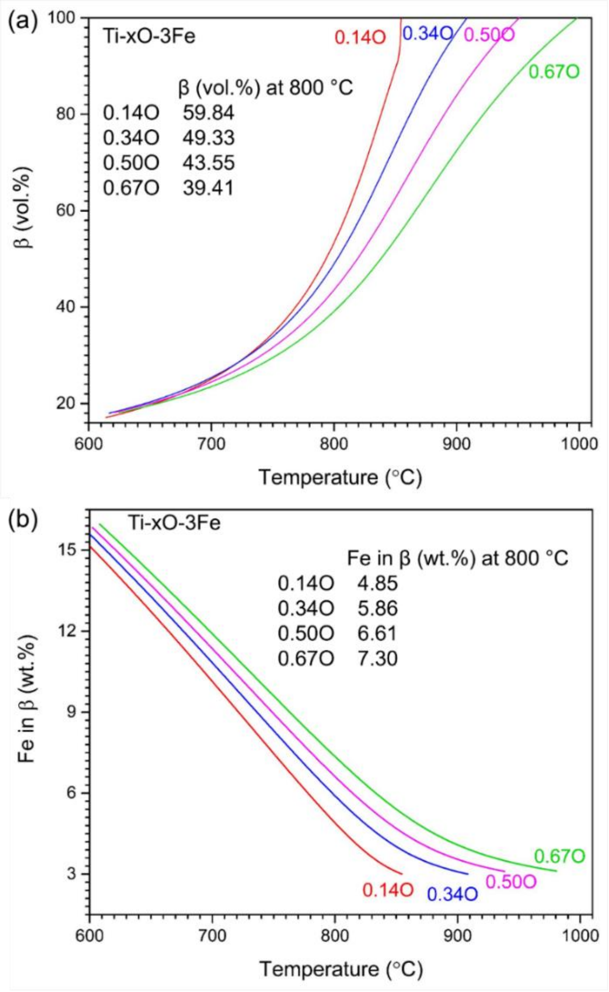

Supplementary Fig. 18 CompuTherm predictions of the β-phase volume fraction and iron content by Pandat^{TM} [3]. (a) Equilibrium volume fraction of the β-phase and (b) the Fe content in the β-phase in each Ti-O-Fe alloy at 800 °C. Increasing the O content results in an increase in the Fe content of the β-phase due to the reduced volume fraction of the β-phase.

Supplementary Note 6

Potential applications and implications

6.1 Potential applications

1) Biomedical sector

Our Ti-O-Fe alloys are biocompatible (containing no toxic elements) and therefore suitable for making both implantable medical devices and surgical and laboratory tools. We take bone fixture plates and reconstruction plates as examples (two classes of medical devices in mass production). Their typical dimensions (thickness: 1.2-3 mm, width: 8-15 mm; length: 50-150 mm or longer) are close to our coupons. They are perforated plates (screw holes or slots). Due to the lack of sufficient ductility and strength, coupled with stress concentration at the edges of these holes, fracture of mill-annealed Ti-6Al-4V bone fixation plates occurs from time to time. Our highly ductile ($\varepsilon_f \geq 20\%$), strong (UTS ≥ 1000 MPa), similarly stiff (E = 110 GPa) Ti-0.35O-3Fe plates with improved biocompatibility manufactured in net shapes by AM can be ideal replacements.

2) Aerospace, marine, defence, chemical processing, pulp and paper production (where austenitic stainless steels only last for 2~3 months) sectors

We limit our predictions to non-fatigue critical applications at this point of time.

Due to their excellent tensile properties in the as-fabricated state, we envisage that these simple Ti-O-Fe alloys, when manufactured in net or near-net shapes by DED, will be attractive and competitive for a broad range of room temperature structural applications that currently use Ti-6Al-4V or ATI 425® or Ti-3Al-2.5V across various sectors. In addition, since our as-fabricated Ti-0.35O-3Fe alloy has already reached the tensile properties of cold-rolled and hot-rolled ATI 425® for ballistic applications, it may be evaluated for ballistic applications as well. Also, they do offer some cost advantages (see below).

3) A potential game changer for off-grade sponge Ti and high-oxygen scrap Ti

The production of sponge Ti through the Kroll process is the basis of the Ti industry. Sponge Ti is produced in the form of sponge lumps (6-15 metric tons per lump). The quality varies wildly from the surface to the centre of each sponge lump by the Fe and O content (and also the residual

Cl content). Off-grade sponge Ti contains > 0.3%O and > 0.4%Fe (e.g. by the National Standard GB/T 2524-2010 of China – China is a major producer and supplier of sponge Ti).

Off-grade sponge Ti comes from the outer layer of each sponge Ti lump (in contact with the steel vessel during the Kroll process) and the sponge scrap generated in the separation process of each sponge lump (each sponge lump will be separated and crushed into small sponge particles of ≤ 25.4 mm). Sponge Ti particles are easy to oxidise due to their large surface area. The Fe content in the off-grade sponge Ti can reach 1.5 wt.%Fe[31,32] while the O content can reach 0.5%.

The volume of the off-grade sponge Ti accounts for **10–20%** of the total sponge production[32-34] (an average of 10% of the sponge production is reasonable). Due to their high O and Fe content, this substantial amount of off-grade sponge Ti is currently used as the raw material of ferro-titanium for the steel industry[32-34] or even to make fireworks.

Sponge titanium production is extremely energy-intensive (423 GJ/ton)[35], which is about 5-10 times the energy consumption of primary aluminium production (which is already considered energy-intensive). It is therefore important to revitalize these off-grade sponge Ti materials.

These off-grade sponge Ti materials are ideal for the production of the Ti-O-Fe alloys developed in this work by DED with high tensile ductility and high strength in net or near-net shapes for advanced applications.

The development we have made in this work provides *a potential approach* to the use of these off-grade sponge Ti materials, which (made into powder) are ideal for the production of the Ti-O-Fe alloys by DED to offer high tensile ductility and high strength in net or near-net shapes for advanced applications. It will turn them into high-grade titanium alloys while contributing to the reduction of the carbon footprint of titanium. This can open up a new chapter for the sponge titanium industry.

Additionally, high-oxygen scrap CP-Ti Grade 3 and Grade 4 can be used in the same way for much higher value creation.

6.2 Implications for sectors other than AM

With a focus on net-shape manufacturing, the findings of this study are best applied to the metal AM sector. Without considering net-shape manufacturing (high buy-to-fly ratio), we envisage that

our $\alpha-\beta$ Ti-O-Fe alloys can be manufactured with significant tensile properties through the combination of PM, elaborate thermo-mechanical processing and machining.

First, ingots of Ti-O-Fe alloys can be made from hydride-dihydride (HDH) high-oxygen Ti powder with Fe powder by cold isostatic pressing (CIP) and sintering. Second, through β-field forging + multi-axial $\alpha-\beta$ field forging + extrusion/rolling, the pre-sintered Ti-O-Fe alloy ingots can be manufactured into billets or plates with fine equiaxed β grains (20-40 μm, much finer than the as-deposited, article file **Fig. 1d-1g**). Finally, heat treatments could be explored to produce fine and short $\alpha-\beta$ lamellae in the fine equiaxed β grains. These Ti-O-Fe microstructures are expected to possess significant tensile properties. Machining will be the ultimate net-shape formation process. The allowed use of high-oxygen HDH Ti powders (low value powder) based on this work is a significant advantage.

Integrating alloy design with AM process design via high-fidelity simulations has allowed us to overcome a significant metallurgical challenge faced by conventional manufacturing — our new class of $\alpha-\beta$ Ti-O-Fe alloys exhibit outstanding tensile properties using the inexpensive and abundant elements O and Fe. Critically, we have demonstrated a wide processing window for the AM of these new alloys. Their simple chemistry and high printability set the scene for a significant disruption in the metallurgy and manufacturing of α-β titanium alloys. Our study could serve as a pathfinder for new directions in alloy development that harness the power of our approach of coupling alloy design and AM process design.

From a titanium alloy design perspective, the excellent tensile properties of $\varepsilon_f = 21.9 \pm 2.2\%$ and $\sigma_{UTS} = 1034 \pm 9$ MPa (E = 112 GPa) obtained for the as-fabricated Ti-0.35O-3Fe alloy demonstrate the high potential of these Ti-O-Fe alloys as unexplored ductile and strong $\alpha-\beta$ Ti alloys. The Ti-0.5O-3Fe alloy ($\varepsilon_f = 9.0 \pm 0.5\%$, $\sigma_{UTS} = 1194 \pm 8$ MPa and E = 120 GPa, without optimisation) could be further processed as the Ti-0.35O-3Fe alloy to obtain much improved properties.

This work offers a promising approach to revitalise large amounts of off-grade sponge Ti (which is sponge Ti-O-Fe) and scrap high-oxygen Ti. These materials could be utilized as feedstock for powder production for AM. Sponge Ti production is highly energy-intensive (5-10 times that of primary aluminium production). Utilising off-grade sponge Ti represents a revitalisation with significant economic and environmental benefits. The same is expected for similarly produced off-grade sponge zirconium (Zr).

Oxygen embrittlement occurs not only in HCP and BCC Ti, but also in other BCC metals (e.g. Nb[36] and Mo[37]), presenting a significant metallurgical challenge. However, here we reveal a unique distribution of oxygen in these AM-fabricated $\alpha-\beta$ Ti-O-Fe alloys. The high tensile ductility of these high-oxygen Ti-O-Fe alloys ($\varepsilon_f = 21.9 \pm 2.2\%$) provides a template for future interstitial engineering in AM-fabricated alloys.

We also discuss the pathfinding potential of this study. For example, zirconium (Zr), exhibits similar physical metallurgy to Ti, and similar metallurgical design approaches could be explored for the development of a new class of strong and ductile $\alpha-\beta$ Zr-O-Fe alloys.

Moreover, a new interstitial engineering opportunity could arise from the results demonstrated here through AM. For instance, similar to oxygen, nitrogen embrittlement also occurs in Ti. As a result, N is tightly controlled to ≤ 0.05%N by conventional manufacturing (≤ 0.05%N), even though N is more potent than O as both α-phase stabiliser and α-phase strengthener.

We have shown that the unique partitioning of oxygen and iron in the two phases (α and β) is fundamental to the success of these Ti-O-Fe alloys (a suitable β-phase volume fraction and the suppression of β-flecks are also important). Following this pathfinding strategy, systematic DFT calculations and predictions could be performed to map out the combination of N with each β-stabiliser (Mo, V, Cr, Fe, Mn, Ni, Co, Nb, Ta, and W) for similar partitioning in the α and β phases. These combinations could lead to the design of potentially attractive new $\alpha-\beta$ titanium alloys. The same approach could be applied to nitrogen in zirconium.

Therefore, this work is not only highly significant in terms of our report of a new class of strong and ductile $\alpha-\beta$ Ti-O-Fe alloys with unique partitioning of O and Fe, but also it offers a potential pathfinding approach to various other new alloy systems.

Supplementary Note 7

Repeatability of tensile stress-strain curves

In this section, we briefly analyse the repeatability issue of the tensile stress-strain curves produced in this work (Extended Data Fig. 2) by focusing on the Ti-0.14O-3.23Fe alloy, whose tensile stress-strain curves displayed the widest gap. Let us refer to Table X1.3 of ASTM E8/E8M-21 (Standard Test Methods for Tension Testing of Metallic Materials), reproduced below, and use it as a starting point.

TABLE X1.3 Precision Statistics—0.2 % Yield Strength, MPa [ksi]

Material	X	s_r	s_r/X, %	s_R	s_R/X, %	r	R
EC-H19	158.4 [22.98]	3.3 [0.47]	2.06	3.3 [0.48]	2.07	9.2 [1.33]	9.2 [1.33]
2024-T351	362.9 [52.64]	5.1 [0.74]	1.41	5.4 [0.79]	1.49	14.3 [2.08]	15.2 [2.20]
ASTM A105	402.4 [58.36]	5.7 [0.83]	1.42	9.9 [1.44]	2.47	15.9 [2.31]	27.8 [4.03]
AISI 316	481.1 [69.78]	6.6 [0.95]	1.36	19.5 [2.83]	4.06	18.1 [2.63]	54.7 [7.93]
Inconel 600	268.3 [38.91]	2.5 [0.36]	0.93	5.8 [0.85]	2.17	7.0 [1.01]	16.3 [2.37]
SAE 51410	967.5 [140.33]	8.9 [1.29]	0.92	15.9 [2.30]	1.64	24.8 [3.60]	44.5 [6.45]
		Averages:	1.35		2.32		

In this table, X is the mean yield strength ($\sigma_{0.2}$) in MPa, S_r is the repeatability standard deviation in MPa, and ***r* is the 95% repeatability limit in MPa** ($r = 2.8 \times S_r$, by the statistical theory[38]). For each test material, if the absolute difference in yield strength (X) between any two repeatability tests (same operator, equipment, environment, and test coupons) is less than the corresponding r value listed in the table, then the repeatability is acceptable.

The ratio of S_r to X for tensile tests typically falls within 1-2% as listed in Table X1.3, supported by the literature data. Hence, as an estimate, we can use $r = 2.8 \times S_r = 2.8 \times (0.01\text{-}0.02)X$. We take $r = 2.8 \times 0.02X$. According to our Extended Data Table 1, for Ti-0.14O-3.23Fe, we have r ($\sigma_{0.2}$) = 41.7 MPa where X = 744 MPa and r (σ_{UTS}) = 49.6 MPa where X = 886 MPa. The maximum absolute difference in $\sigma_{0.2}$ for this alloy (see Extended Data Fig. 2a) is 32 MPa **<** r = 41.7 MPa, while the maximum absolute difference in σ_{UTS} is 36 MPa **<** r = 49.6 MPa. Therefore, although the stress-strain curves for Ti-3.23Fe-0.14O do look less repeatable (there is a good reason. See below) than other Ti-Fe-O alloys, the results meet the expected repeatability limit requirements. Nonetheless, the S_r value should be as small as possible.

As shown in Supplementary Note 2, the influence of significant chemical inhomogeneity can be excluded. To further substantiate this point, we have deposited the Ti-185 (Ti-1Al-8V-5Fe) alloy using the same DED system with top-quality pre-alloyed Ti-185 powder ordered from a commercial supplier, produced using a high-speed (17,000 rpm) plasma rotating electrode process

(PREP). The PREP produces highly spherical powder with negligible internal porosity and gas. A similar degree of discrepancy, excluding the influence of chemical inhomogeneity, was observed in the tensile stress-strain curves, shown in Supplementary Fig. 19.

Therefore, we attribute the wider than expected discrepancy in tensile properties for the Ti-0.14O-3.23Fe alloy mainly to its grain structure. The Ti-0.14O-3.23Fe alloy has the coarsest, most irregular prior-β grains (250 ± 12 μm in width, Fig. 1d) due to its **lowest O content** (O promotes the CET in these alloys). No lack-of-fusion defects are observed from the fracture surface of this particular specimen. Other conditions are all similar.

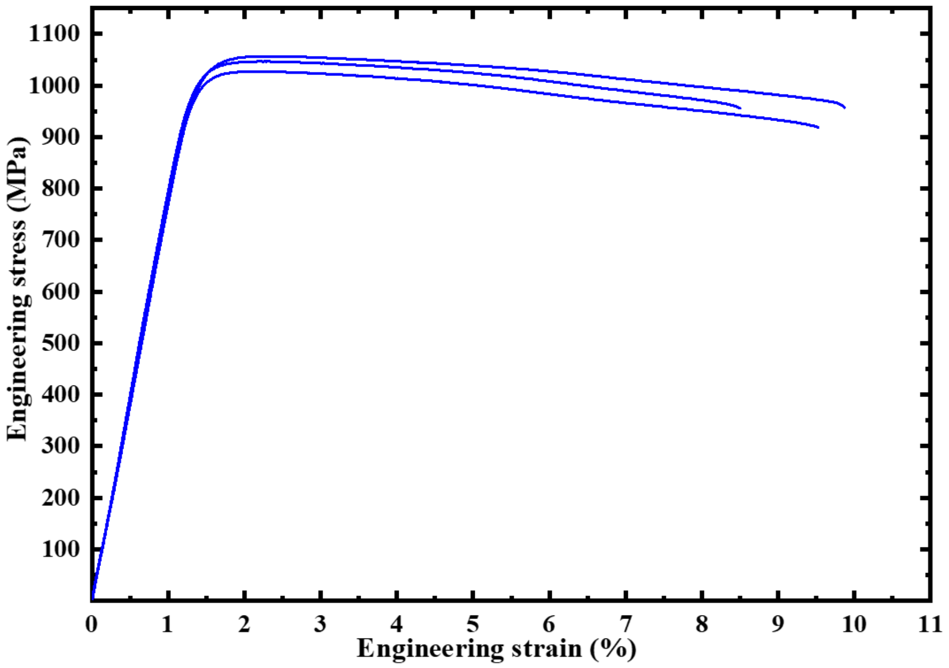

Supplementary Fig. 19 Tensile stress-strain curves of Ti-1Al-8V-5Fe (wt.%) fabricated using the same DED system with high-quality pre-alloyed PREP powder. A larger than expected discrepancy in the tensile stress-strain curves was observed, excluding the influence of chemical inhomogeneity. These tensile stress-strain curves are still within the repeatability limit according to ASTM E8/E8M – 21.

In addition, we also plotted the true stress (σ_{true}) versus true strain ($\varepsilon_{\mathrm{true}}$) curves corresponding to Fig. 2 using the formulae $\varepsilon_{\mathrm{true}} = \ln(1 + \varepsilon_{\mathrm{eng}})$ and $\sigma_{\mathrm{true}} = \sigma_{\mathrm{eng}}(1 + \varepsilon_{\mathrm{eng}})$ [39], valid up to the point where $\mathrm{d}\sigma_{\mathrm{eng}}/\mathrm{d}\varepsilon_{\mathrm{eng}} = 0$ or $\sigma = \sigma_{\mathrm{UTS}}$. For negligible necking (observed in this work, Supplementary Fig. 13), the conversion may be extended beyond the σ_{UTS} as an approximation. Supplementary Fig. 20 shows the curves after conversion.

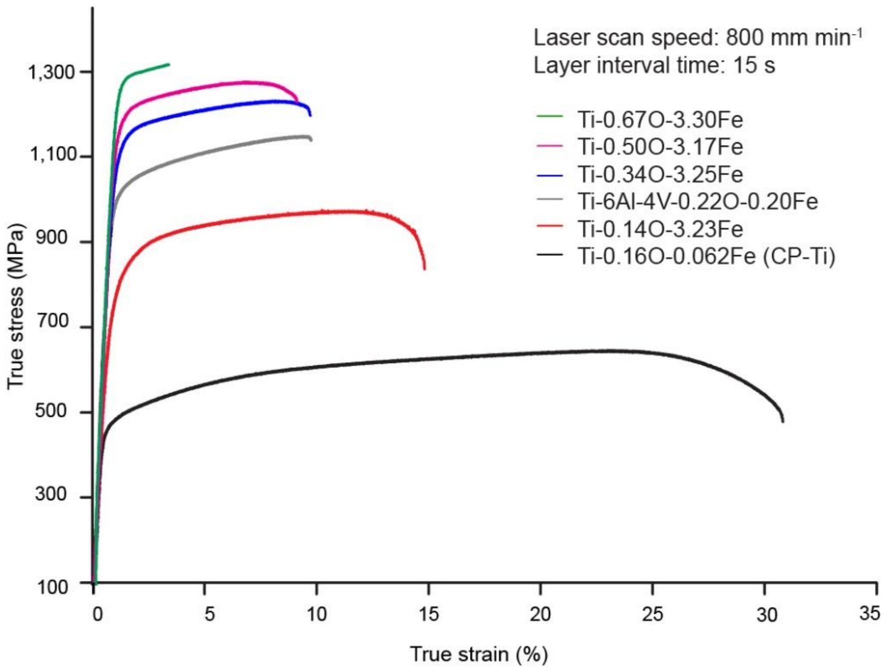

Supplementary Fig. 20 Calculated true stress–true strain curves from Fig. 2 using $\varepsilon_{\text{true}} = \ln(1 + \varepsilon_{\text{eng}})$ and $\sigma_{\text{true}} = \sigma_{\text{eng}}(1 + \varepsilon_{\text{eng}})$ [39]. Strictly, this expression is valid only up to the point where $\text{d}\sigma_{\text{eng}}/\text{d}\varepsilon_{\text{eng}} = 0$ or $\sigma = \sigma_{\text{UTS}}$. We wish to unambiguously emphasise that inputs to these simple expressions for true stress and true strain as charted here are not strictly valid for engineering stress and strain inputs beyond $\text{d}\sigma_{\text{eng}}/\text{d}\varepsilon_{\text{eng}} = 0$, or the σ_{UTS}. The case for extending these true stress-true strain plots beyond the σ_{UTS} (as plotted above) can be made for situations where, as observed in this work (Supplementary Fig. 13), there is negligible necking. We recommend all quantitative assessments of our tensile properties to be made from the raw engineering stress–engineering strain data provided in Extended Data Fig. 2.

References for Supplementary Information

1 Lütjering, G. & Williams, J. C. *Titanium*. (Springer Berlin, Heidelberg, 2007).

2 Polmear, I., StJohn, D., Nie, J.-F. & Qian, M. *Light Alloys: Metallurgy of the Light Metals*. (Butterworth-Heinemann, 2017).

3 Cao, W. *et al.* PANDAT software with PanEngine, PanOptimizer and PanPrecipitation for multi-component phase diagram calculation and materials property simulation. *Calphad* **33**, 328-342 (2009).

4 Haubrich, J. *et al.* The role of lattice defects, element partitioning and intrinsic heat effects on the microstructure in selective laser melted Ti-6Al-4V. *Acta Mater.* **167**, 136-148, (2019).

5 Zhang, J. *et al.* Designing against phase and property heterogeneities in additively manufactured titanium alloys. *Nat. Commun.* **13**, 4660, (2022).

6 Kumar, S. *et al.* Role of thermo-mechanical gyrations on the α/β interface stability in a Ti6Al4V AM alloy. *Scr. Mater.* **204**, 114134, (2021).

7 Tan, X. *et al.* Graded microstructure and mechanical properties of additive manufactured Ti–6Al–4V via electron beam melting. *Acta Mater.* **97**, 1-16, (2015).

8 Sridharan, N. *et al.* On the potential mechanisms of β to α′ + β decomposition in two phase titanium alloys during additive manufacturing: a combined transmission Kikuchi diffraction and 3D atom probe study. *J. Mater. Sci.* **55**, 1715-1726, (2020).

9 Tan, X. *et al.* Revealing martensitic transformation and α/β interface evolution in electron beam melting three-dimensional-printed Ti-6Al-4V. *Sci. Rep.* **6**, 26039, (2016).

10 Martin, T. L. *et al.* Insights into microstructural interfaces in aerospace alloys characterised by atom probe tomography. *Mater. Sci. Technol.* **32**, 232-241, (2016).

11 Shamblen, C. E. Minimizing beta flecks in the Ti-17 alloy. *Metall. Mater. Trans. B* **28**, 899-903 (1997).

12 Mitchell, A., Kawakami, A. & Cockcroft, S. Beta fleck and segregation in titanium alloy ingots. *High Temp. Mater. Process.* **25**, 337-349 (2006).

13 Zeng, W. & Zhou, Y. Effect of beta flecks on mechanical properties of Ti–10V–2Fe–3Al alloy. *Mater. Sci. Eng. A* **260**, 203-211 (1999).

14 Clyne, T. W. & Kurz, W. Solute redistribution during solidification with rapid solid state diffusion. *Metall. Trans. A* **12**, 965-971 (1981).

15 Kurz, W. & Trivedi, R. Rapid solidification processing and microstructure formation. *Mater. Sci. Eng. A* **179-180**, 46-51 (1994).

16 Pinomaa, T., Laukkanen, A. & Provatas, N. Solute trapping in rapid solidification. *MRS Bull.* **45**, 910-915, (2020).

17 Song, R., Dai, F. & Wei, B. Dendritic growth and solute trapping in rapidly solidified Cu-based alloys. *Sci. China Phys. Mech.* **54**, 901-908, (2011).

18 Zhou, Q., Zhang, X. Z., Tang, H. P. & Qian, M. Electron beam additively manufactured Ti–1Al–8V–5Fe alloy: In-situ precipitation hardening, tensile properties and fracture characteristics. *Mater. Sci. Eng. A* **865**, 144639, (2023).

19 Broderick, T. F., Jackson, A. G., Jones, H. & Froes, F. H. The effect of cooling conditions on the microstructure of rapidly solidified Ti-6Al-4V. *Metall. Trans. A* **16**, 1951-1959, (1985).

20 Kozieł, T. Estimation of cooling rates in suction casting and copper-mould casting processes. *Arch. Metall. Mater.* **60**, 767-771 (2015).

21 Srivastava, R. M., Eckert, J., Löser, W., Dhindaw, B. K. & Schultz, L. Cooling rate evaluation for bulk amorphous alloys from eutectic microstructures in casting processes. *Mater. Trans.* **43**, 1670-1675 (2002).

22 Liu, C. Investigating the effect of cooling rate on the secondary dendrite arm spacing in titanium alloys. Engineering Thesis, The University of Queensland (2020).

23 Stefanescu, D. M. *Science and engineering of casting solidification*, p. 23 (Springer, 2015).

24 Kim, D.-H. & Lee, C.-M. Experimental investigation on machinability of titanium alloy by laser-assisted end milling. *Metals* **11**, 1552 (2021).

25 Wu, Q. *et al.* Effect of molten pool size on microstructure and tensile properties of wire arc additive manufacturing of Ti-6Al-4V alloy. *Materials* **10**, 749 (2017).

26 Mok, S. H., Bi, G., Folkes, J. & Pashby, I. Deposition of Ti–6Al–4V using a high power diode laser and wire, Part I: Investigation on the process characteristics. *Surf. Coat. Technol.* **202**, 3933-3939, (2008).

27 Tran, H. S. *et al.* 3D thermal finite element analysis of laser cladding processed Ti-6Al-4V part with microstructural correlations. *Mater. Des.* **128**, 130-142, (2017).

28 Wang, H., Yang, S. & Wei, B. Density and structure of undercooled liquid titanium. *Chin. Sci. Bull.* **57**, 719-723 (2012).

29 Ozawa, S., Kudo, Y., Kuribayashi, K., Watanabe, Y. & Ishikawa, T. Precise density measurement of liquid titanium by electrostatic levitator. *Mater. Trans.* **58**, 1664-1669 (2017).

30 *Titanium C.P. Grade 2 Datasheet* https://www.upmet.com/sites/default/files/datasheets/cp-grade-2.pdf (United Performance Metals).

31 Osipenko, A. B. Development of technology of obtaining raw materials for titanium alloys made of off-grade titanium sponge. *EasternEuropean J. Enterp. Technol.* **4**, 28-32, (2015).

32 Marui, Y., Kinoshita, T. & Takahashi, K. Development of a titanium material by utilizing off-grade titanium sponge. *SAE Tech. Pap.* **32**, 1816, (2002).

33 Takeda, O., Ouchi, T. & Okabe, T. H. Recent progress in titanium extraction and recycling. *Metall. Mater. Trans. B* **51**, 1315-1328, (2020).

34 Taninouchi, Y. K., Hamanaka, Y. & Okabe, T. H. Titanium recycling process based on A chlorination reaction utilizing chloride waste in *Proceedings of the 13th World Conference on Titanium* 165-170 (2016).

35 Gao, F. *et al.* Environmental impacts analysis of titanium sponge production using Kroll process in China. *J. Clean. Prod.* **174**, 771-779 (2018).

36 Yang, P.-J. *et al.* Mechanism of hardening and damage initiation in oxygen embrittlement of body-centred-cubic niobium. *Acta Mater.* **168**, 331-342 (2019).

37 Wang, Z.-Q. *et al.* Suppressing effect of carbon on oxygen-induced embrittlement in molybdenum grain boundary. *Comput. Mater. Sci.* **198**, 110676 (2021).

38 Repeatability and reproducibility. *Stats Book* http://pcool.dyndns.org:8080/statsbook/?page_id=835.

39 Dieter, G. E. & Bacon, D. *Mechanical metallurgy*. (New York: McGraw-hill, 1988, pp. 72-75).
